# Depth‐Dependence Changes in Soil Stoichiometry in China's Croplands Over the Past Four Decades

**DOI:** 10.1002/advs.202506489

**Published:** 2025-09-25

**Authors:** Xiaodong Sun, Zhenghu Zhou, Yiqi Luo, Qingzhu Gao, Hu Li, Shuo Liu, Minggang Xu, Yu'e Li, Andong Cai

**Affiliations:** ^1^ Institute of Environment and Sustainable Development in Agriculture Chinese Academy of Agricultural Sciences Beijing 100081 China; ^2^ Soil Health Laboratory of Shanxi Province Institute of Eco‐environment and Industrial Technology Shanxi Agricultural University Taiyuan 030031 China; ^3^ Center for Ecological Research Northeast Asia Biodiversity Research Center Northeast Forestry University Harbin 150040 China; ^4^ School of Integrative Plant Science Cornell University Ithaca NY 14853 USA; ^5^ Institute of Agricultural Resources and Regional Planning Chinese Academy of Agricultural Sciences Beijing 100081 China

**Keywords:** agroecosystems, management practice, soil profiles, soil stoichiometry, temporal dynamics

## Abstract

Soil carbon (C), nitrogen (N), and phosphorus (P) stoichiometry is an integrative metric that reflects the balance and limiting relationships among soil elements. However, how climate change and nutrient inputs affect cropland C:N:P ratios along soil profiles at large scales remains largely unknown. Here, variations in soil C:N:P ratios across a 0–100 cm soil profile in China's croplands is assessed, based on repeated measurements at 305 resampling sites in the 1980s and 2023, totaling 6405 soil profiles. Over the past four decades, the mean soil C:N ratio across the entire profile increases by 20.18%, the C:P ratio increases by 4.49%, while the N:P ratio decreases by 9.02%. Spatially, changes in C:N and N:P ratios tend to weaken with increasing latitude. These changes exhibit clear depth‐dependent patterns. The soil C:N ratios increase across the entire soil profile, while the C:P and N:P ratios increase in the topsoil (0–40 cm) but decrease in the subsoil (40–100 cm), primarily driven by organic C and nutrient inputs, influenced by initial soil conditions and climate. The results highlight the depth‐dependent changes in soil stoichiometry in response to long‐term management practices and their implications for ecosystem services.

## Graphical abstract

This study reveals depth‐dependent changes in soil C:N:P ratios across Chinese croplands over four decades. Changes in stoichiometry vary markedly between topsoil and subsoil, with initial soil C:N:P ratios playing a key role in determining their trajectories. Long‐term carbon and nutrient inputs and climate variability further influence these changes across space and depth.

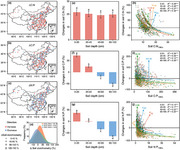

## 1 Introduction

The biogeochemical cycles of carbon (C), nitrogen (N), and phosphorus (P) exhibit biological coupling from the molecular level to the global scale, owing to organisms that catalyze metabolic reactions with strict elemental proportions.^[^ ^1^ ^]^ Consequently, the stoichiometric C:N:P ratios of soils serve as coupling indices that quantitatively reflect the biological processes and nutrient cycling in terrestrial ecosystems.^[^ ^2^, ^3^ ^]^ Despite their robust coupling, the biogeochemical cycles of soil C, N, and P have exhibited weakened relationships or independent variations under global climate change and unregulated management practices due to the disparate levels of control applied to these elements by ecological and chemical mechanisms, a phenomenon referred to as decoupling, whereby changes in stoichiometric C:N:P ratios are regarded as its manifestations and consequences that reflect altered nutrient balances.^[^ ^4^, ^5^, ^6^, ^7^ ^]^ Changes in soil C:N:P ratios have led to the dissociation of nutrients in plants that form the basis of food chains, as well as negative effects on terrestrial ecosystem service functions.^[^ ^1^ ^]^ Therefore, accurately quantifying the dynamics of soil C:N:P ratios is essential for predicting the responses of terrestrial ecosystems to global climate change, particularly at regional scales.

The magnitude of changes in soil C:N:P ratios remains a subject of intense debate. A comprehensive understanding of regional soil C:N:P dynamics requires extensive laboratory evaluations and precise initial data collected over decades. To address these challenges, methods such as space‐for‐time substitution, literature compilation, and modeling simulations have been increasingly utilized. However, each of these approaches has notable limitations.^[^ ^8^, ^9^, ^10^ ^]^ The space‐for‐time substitution method overlooks the spatial heterogeneity of initial soil C:N:P ratios, mismatches in time scales, and the diversity of environmental variables.^[^ ^11^ ^]^ Literature compilation, which relies on published data, is hampered by unstandardized soil sampling, inconsistent soil depths, time periods, and analytical techniques.^[^ ^12^ ^]^ Soil models, essential for evaluating and predicting soil C, N, and P dynamics, face uncertainties due to differences in model structures, baseline parameters, and calibration and validation techniques at regional and global scales.^[^ ^13^ ^]^ Collectively, these methods rely on assumptions and cannot accurately capture soil C:N:P dynamics. In contrast, direct field measurements of soil C, N, and P at the same sites over different time intervals provide a reliable approach that overcomes the limitations of space‐for‐time substitution, literature compilation, and modeling simulations, yielding more accurate and dependable soil C:N:P data.

Most studies on soil C:N:P dynamics have focused on surface soils due to their accessibility and relevance to real‐time ecological processes.^[^ ^4^, ^14^, ^15^ ^]^ However, the responses of soil C:N:P ratios to environmental variables vary with soil depth.^[^ ^3^ ^]^ Investigating subsoil C:N:P dynamics is crucial for understanding soil nutrient cycling and interactions, as well as for assessing and preserving ecosystem services, given that subsoils store over 70% of soil C and nutrients.^[^ ^16^ ^]^ Despite this, subsoil C:N:P dynamics have been largely overlooked at regional scales, likely due to two main factors: the traditional assumption that subsoils are inert^[^ ^17^ ^]^ and the specialized equipment and techniques required for subsoil sampling, which incur significant time and economic costs.^[^ ^12^ ^]^ Focusing solely on topsoil for research into soil C:N:P dynamics can translate to significant bias and inaccurate conclusions toward understanding the stoichiometric ratios of the entire soil profile.^[^ ^18^ ^]^ Therefore, comprehensive investigations of C:N:P dynamics should include both topsoil and subsoil to fully comprehend soil C, N, and P cycles.

Widespread organic C inputs (e.g., root stubble, straw, manure) and higher N and P inputs in agroecosystems rapidly induce soil C:N:P imbalances compared with natural ecosystems, which subsequently alter soil ecological functions.^[^ ^19^ ^]^ As an agriculturally intensive nation, China has become the largest contributor to the use of N and P fertilizers to increase crop productivity and meet food demand.^[^ ^20^, ^21^ ^]^ Furthermore, China has implemented straw incorporation policies since 2000 to reduce environmental pollution and improve soil fertility, while also supplying additional C.^[^ ^22^ ^]^ These long‐term agricultural practices and policies have significantly altered soil C:N:P ratios. Fortunately, China's Second National Soil Inventory in the 1980s provided a valuable baseline for soil C, N, and P levels, facilitating a detailed study of soil C:N:P dynamics. For this study, we conducted large‐scale soil resampling at 305 sites in 2023 (spanning ≈25° of latitude and ≈55° of longitude), matching the original 1980s sampling sites. The resampling included 6405 soil profiles and 25620 soil samples across different soil depths (0–20, 20–40, 40–60, and 60–100 cm). To explain the variations in soil C:N:P ratios, we considered various factors, including climate (accumulated temperature (AT) and accumulated precipitation (AP), actual evapotranspiration (AET), temperature seasonality (T_season_), and precipitation seasonality (P_season_), management practices (C, N, and P inputs), and soil properties (pH, texture, bulk density (BD), cation exchange capacity (CEC), and 1980s C:N:P ratios). For clarity, all key variables and abbreviations used in this study are summarized in **Table** **1**.

**Table 1:** Summary of key variables and abbreviations used in this study.

Variable/Abbreviation	Variable definition
SOC (g kg^−1^)	Soil organic carbon concentration
TN (g kg^−1^)	Soil total nitrogen concentration
TP (g kg^−1^)	Soil total phosphorus concentration
SOC absolute change (g kg^−1^)	SOC_2023_ – SOC_1980s_
TN absolute change (g kg^−1^)	TN_2023_ – TN_1980s_
TP absolute change (g kg^−1^)	TP_2023_ – TP_1980s_
SOC relative change (%)	Relative (percentage) change in SOC concentration between the 1980s and 2023
TN relative change (%)	Relative (percentage) change in TN concentration between the 1980s and 2023
TP relative change (%)	Relative (percentage) change in TP concentration between the 1980s and 2023
C:N	Soil C to N molar ratio
C:P	Soil C to P molar ratio
N:P	Soil N to P molar ratio
ΔC:N (%)	Relative change in soil C:N ratio between 1980s and 2023
ΔC:P (%)	Relative change in soil C:P ratio between 1980s and 2023
ΔN:P (%)	Relative change in soil N:P ratio between 1980s and 2023
C:N absolute change	Absolute difference in C:N ratio between 2023 and 1980s (C:N_2023_ – C:N_1980s_)
C:P absolute change	Absolute difference in C:P ratio between 2023 and 1980s (C:P_2023_ – C:P_1980s_)
N:P absolute change	Absolute difference in N:P ratio between 2023 and 1980s (N:P_2023_ – N:P_1980s_)
C_input_ (kg ha^−1^)	Cumulative C inputs from straw, root residues, and manure (1980s–2023)
N_input_ (kg ha^−1^)	Cumulative N inputs from straw, crop residues, and mineral fertilizers (1980s–2023)
P_input_ (kg ha^−1^)	Cumulative P inputs from straw, crop residues, and mineral fertilizers (1980s–2023)
ΔAET (%)	Relative change in actual evapotranspiration between the 1980s and 2023
AT (°C)	Accumulated temperature (1980s–2023)
AP (mm)	Accumulated precipitation (1980s–2023)
T_season_ (°C × 100)	Temperature seasonality (WorldClim Bio4)
P_season_ (%)	Precipitation seasonality (WorldClim Bio15)
ΔBD (%)	Relative change in bulk density between the 1980s and 2023
ΔCEC (%)	Relative change in Cation exchange capacity between 1980s and 2023

## 2 Results

### 2.1 General and Spatial Patterns of Changes in Soil C:N:P

**Figure 1:**
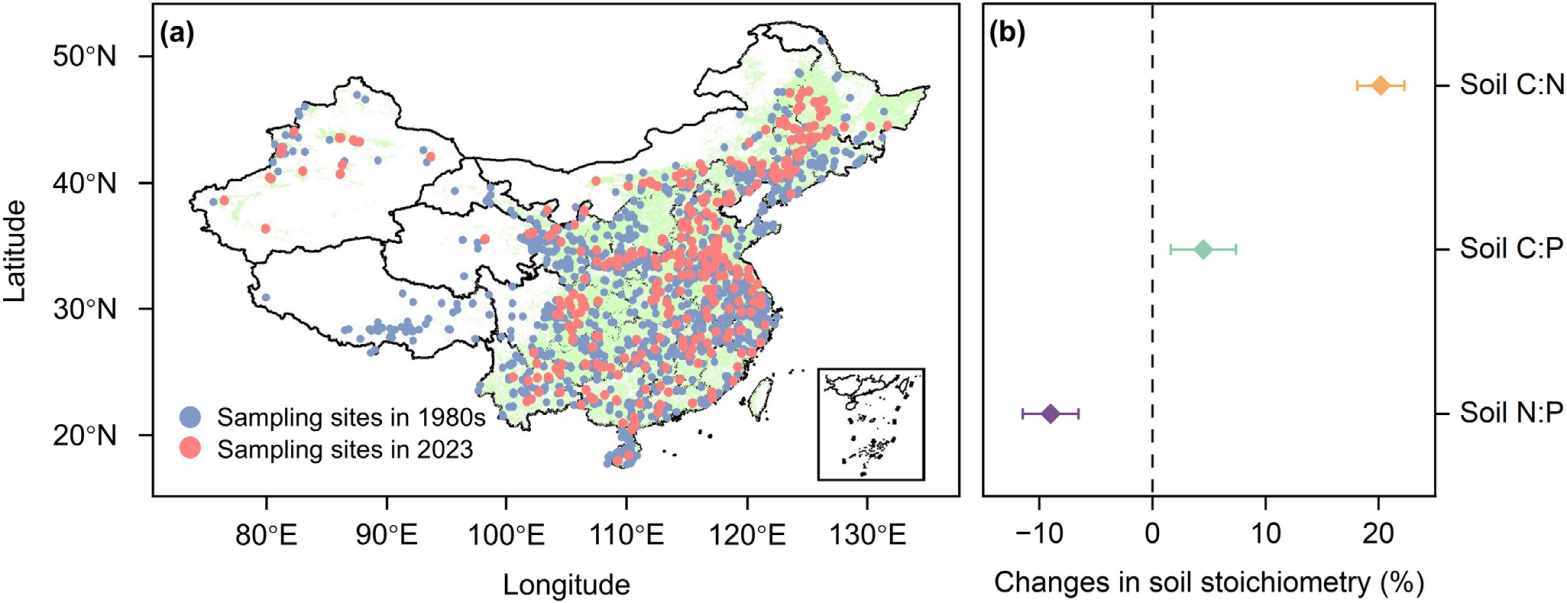
Distribution of sampling sites (a) and soil C:N:P changes (b) in China's cropland soils over the last 40 years. Changes in soil stoichiometry were the relative change rate, referring to changes in the soil C:N, C:P, and N:P in 2023 relative to the 1980s. Error bars represent 95% confidence intervals (CI).

**Figure 2:**
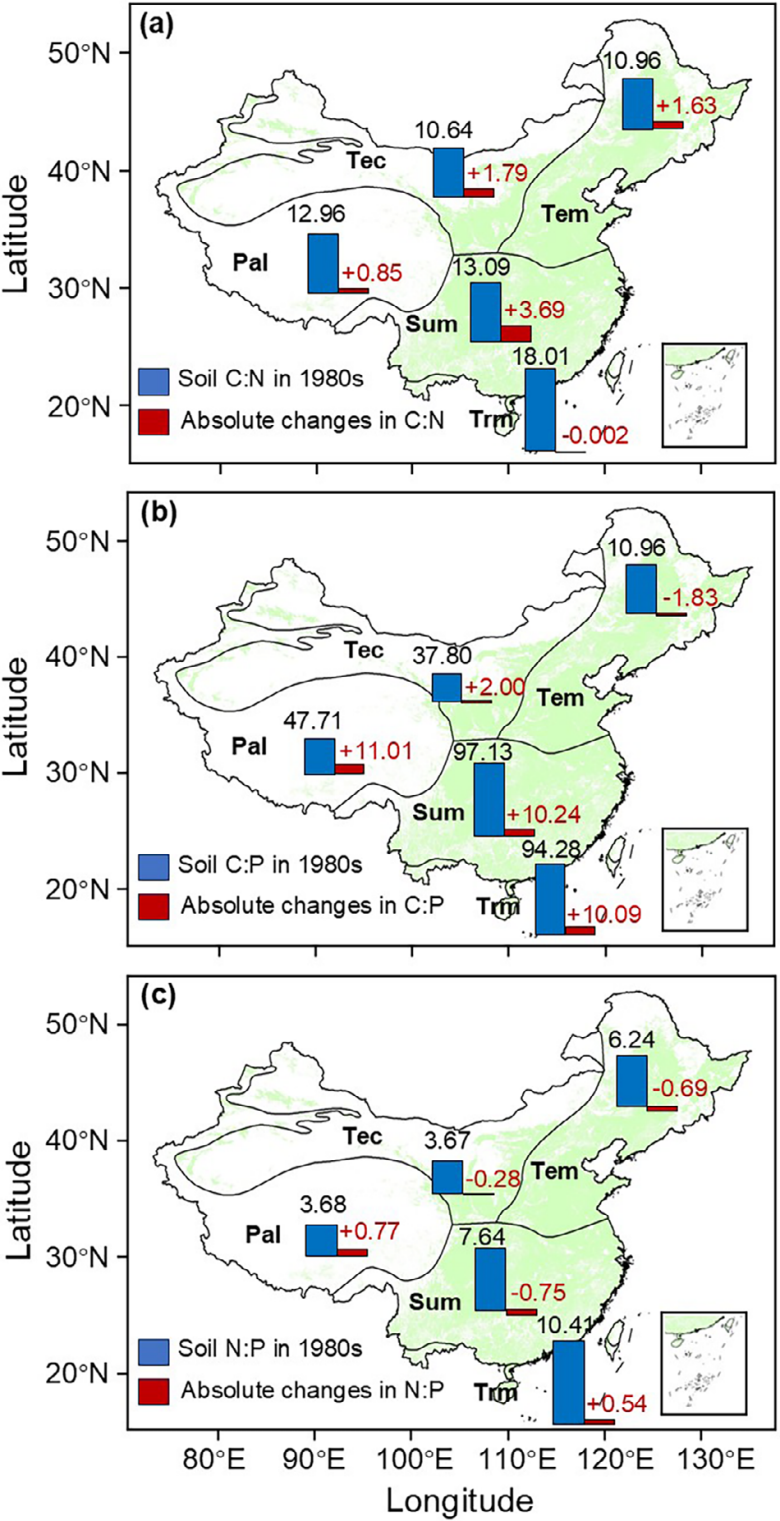
Spatial variations in soil C:N (a), C:P (b), and N:P (c) changes in China's croplands over the last 40 years. Absolute change refers to the difference in soil stoichiometric ratios between 2023 and the 1980s, while relative change expresses this difference as a proportion of the 1980s value. Tec, temperate continental zone; Tem, temperate monsoon zone; Pal, Plateau‐alpine zone; Sum, subtropical monsoon zone; Trm, tropical monsoon zone.

This study represents the first comprehensive assessment of soil C:N:P dynamics across the entire soil profile (0–100 cm) in China's croplands. We conducted repeated measurements of soil C, N, and P at 305 resampling sites in the 1980s and in 2023, covering 6405 soil profiles (**Figure** **1**a). In the 1980s, the initial soil C:N, C:P, and N:P ratios were 11.92, 73.08, and 6.48, respectively (Figure S1, Supporting Information). Over the last four decades, the soil C:N and C:P ratios increased by 20.18% and 4.49%, respectively, while the N:P ratio decreased by 9.02% (Figure 1b). Furthermore, China's croplands have undergone spatially heterogeneous changes in soil stoichiometry (**Figure** **2**). The magnitude of changes in soil C:N and N:P ratios declined with increasing latitude (Figure S2, Supporting Information). Soil C:N ratios increased in most climatic zones, with the largest rise observed in the subtropical monsoon zone (+3.69). Although the tropical monsoon zone had the highest baseline (1980s) C:N ratio, it remained largely unchanged, with a negligible decrease of 0.002. Baseline soil C:P ratios exhibited substantial spatial variation, reaching 97.13 in the subtropical monsoon zone and 94.28 in the tropical monsoon zone, but only 10.96 in the temperate monsoon zone. However, the relative changes in C:P did not differ significantly among climatic zones (Figure S3, Supporting Information). The plateau–alpine zone, which had a relatively low baseline N:P ratio (3.68), exhibited the greatest increase (+0.77), whereas N:P ratios declined in the temperate monsoon, temperate continental, and subtropical monsoon zones. Changes in soil C:N ratios were linked to soil type and parent material, whereas variations in C:P and N:P were not influenced by these factors (Figure S4, Supporting Information).

### 2.2 Depth‐Dependence Changes in Soil C:N:P

**Figure 3:**
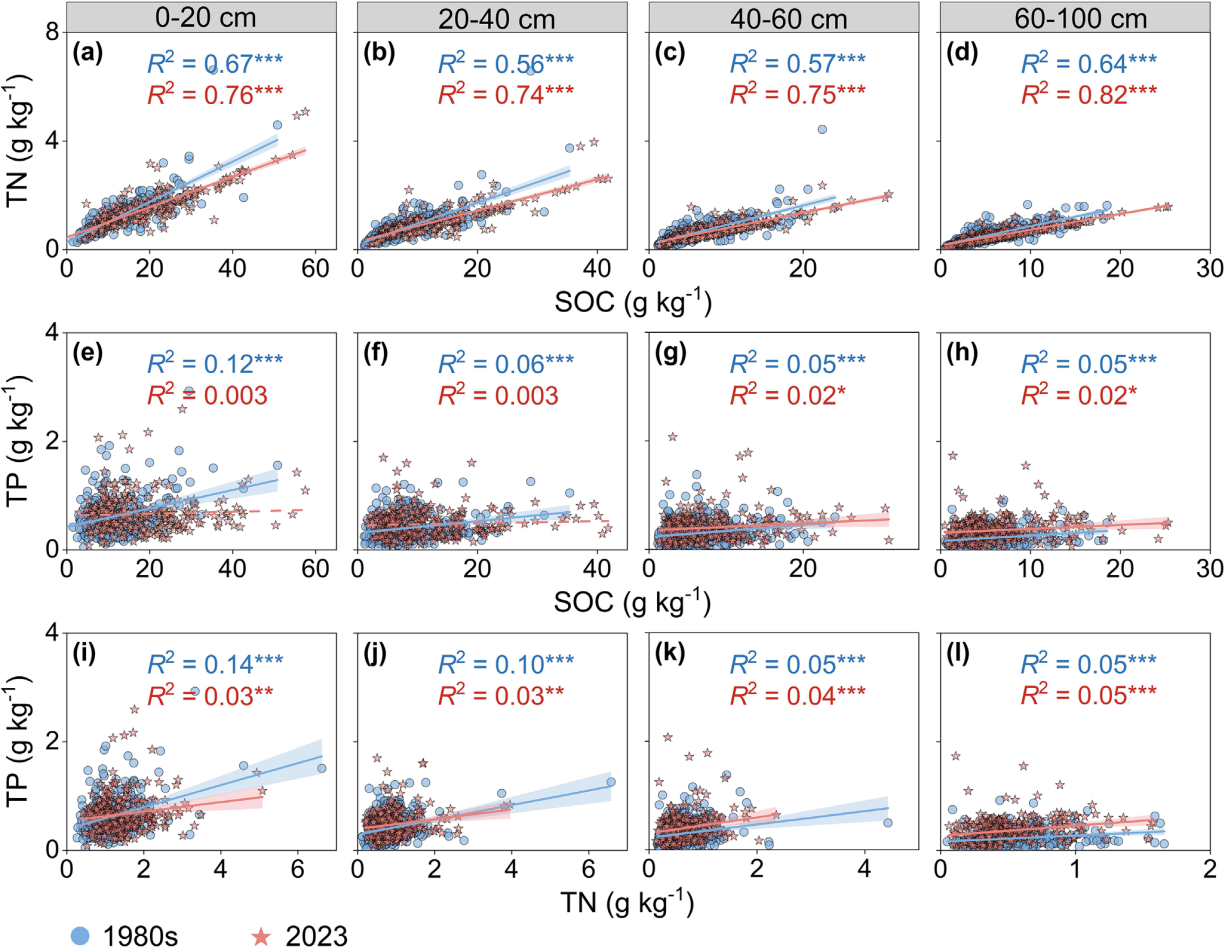
Relationship between soil organic carbon, total nitrogen, and total phosphorus in Chinese croplands over the past 40 years. The figure shows linear relationships between soil organic C (SOC) and total N (TN) (a–d), SOC and total P (TP) (e–h), and TN and TP (i–l) at soil depths of 0–20 cm, 20–40 cm, 40–60 cm, and 60–100 cm. The solid line represents the significant linear regression (*p* < 0.05), and the shading indicates the 95% confidence intervals. ^*^ *p* < 0.05, ^**^ *p* < 0.01, ^***^ *p* < 0.001.

**Figure 4:**
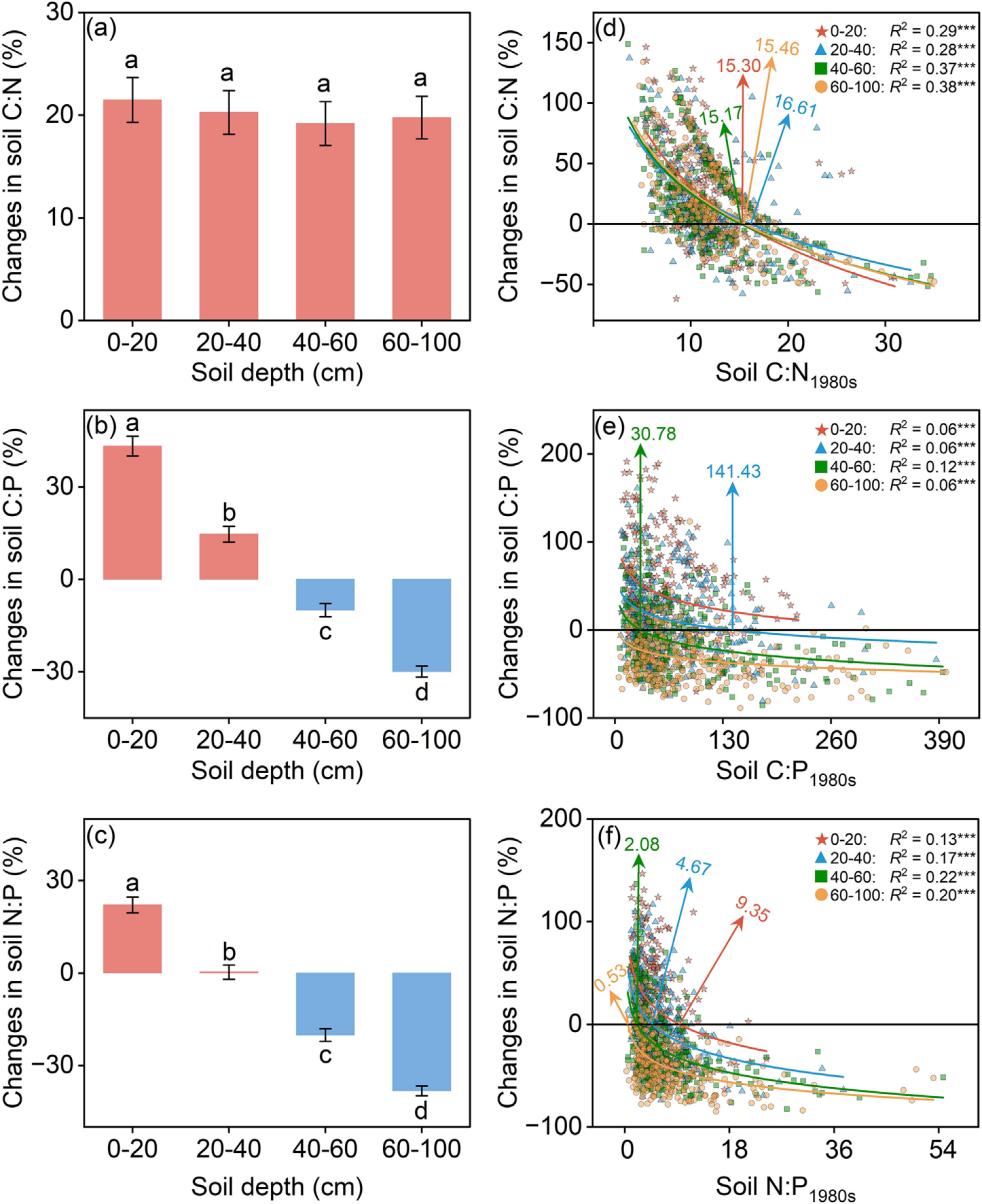
Depth dependence of changes in soil C:N:P stoichiometry in China's croplands over the last 40 years. Changes in soil stoichiometry were the relative change rate, referring to changes in the soil C:N, C:P, and N:P in 2023 relative to the 1980s. a–c) changes in soil C:N (a), C:P (b), and N:P (c) at different depths, different lowercase letters indicate significant differences between soil depths (*p* < 0.05), the error bars represent ± standard errors (SE). d–f) relationship of changes in soil C:N (d), C:P (e), and N:P (f) to the soil nutrients in the 1980s, ^***^ *p* < 0.001.

Over the past four decades, SOC increased consistently across the entire profile (0.75–5.17 g kg^−1^, 21.97–64.98%), and TN also exhibited an overall rise (7.21–39.31%), with the most pronounced increase in the 0–20 cm layer. In contrast, TP showed continuous increases across all depths (0.04–0.14 g kg^−1^, 23.42–89.78%), with relative gains becoming progressively larger in deeper layers (Figure S5, Supporting Information). Additionally, in the 1980s, soil C, N, and P were tightly coupled throughout the entire soil profile (0–100 cm) in croplands, but after more than 40 years, a decoupling among these elements has emerged (**Figure** **3**). Specifically, although C and N remain closely linked, the relationships between C and P, as well as between N and P, have gradually declined, and in the topsoil layer (0–40 cm), the coupling between C and P has disappeared (*R* ^2^ decreased from 0.12 and 0.06 to 0.003, non‐significant; Figure 3e,f). The decoupling among C, N, and P resulted in corresponding changes in C:N:P ratios. For different soil layers, the Δsoil C:N ratios showed no statistically significant differences (**Figure** **4**a). In contrast, the C:P and N:P ratios exhibited marked depth‐dependent changes, increasing in the topsoil but decreasing in the subsoil (40–100 cm) (Figure 4b,c). Specifically, the C:P ratios increased by 43.25% and 14.67% in the 0–20 and 20–40 cm layers, respectively, but decreased by 10.02% and 29.93% in the 40–60 and 60–100 cm layers, respectively. Similarly, the N:P ratio increased by 22.09% and 0.29% in the 0–20 and 20–40 cm layers, respectively, but decreased by 20.16% in both the 40–60 and 60–100 cm layers. These patterns were consistent across various climatic zones, soil types, and parent materials, with notable differences observed in the subsoil (Figures S6–S8, Supporting Information).

For every soil layer, a significant negative logarithmic relationship was observed between the Δsoil C:N:P and soil C:N:P_1980s_ (Figure 4d–f). Notably, the Δsoil C:N:P ratios exhibited distinct thresholds as soil C:N:P_1980s_ increased. Specifically, the thresholds for Δsoil C:N were found within a narrow range of soil C:N_1980s_ values (15.17 to 16.61). For Δsoil C:P, thresholds were identified only in the 20–40 cm (141.43) and 40–60 cm (30.78) soil layers. The Δsoil N:P thresholds varied across soil layers, with values of 9.35, 4.67, 2.08, and 0.53 in the 0–20, 20–40, 40–60, and 60–100 cm layers, respectively. Over the past 40 years, the soil C:P ratio consistently increased in the 0–20 cm layer but decreased in the 60–100 cm layer.

### 2.3 Drivers of Changes in Soil C:N:P

**Figure 5:**
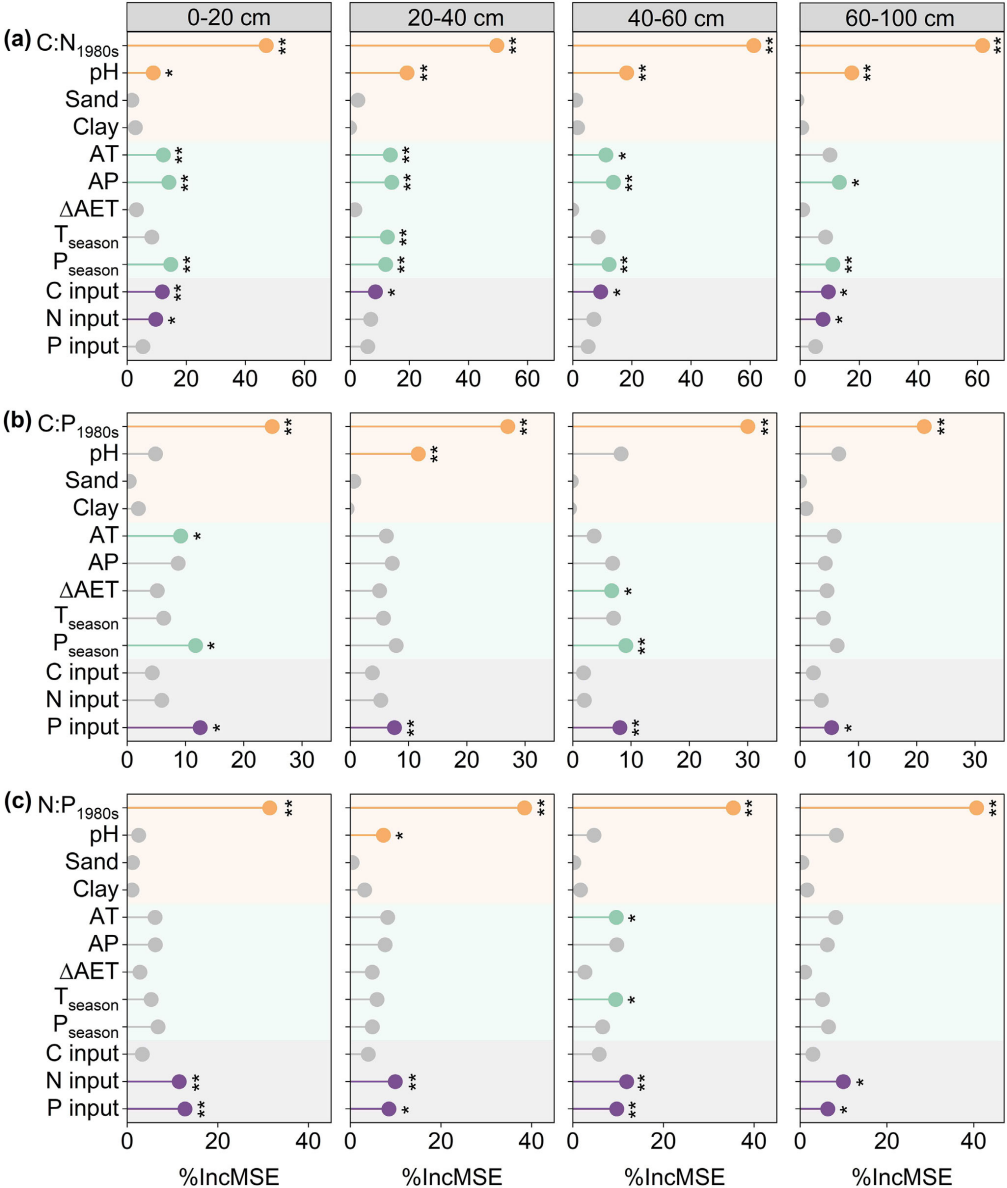
Contribution of environmental variables to changes in soil C:N (a), C:P (b), and N:P (c) stoichiometry by the random forest model. Δsoil C:N, Δsoil C:P, and Δsoil N:P represent the relative change rate, referring to changes in the soil C:N, C:P, and N:P in 2023 relative to 1980s. C:N:P_1980s_, background (1980s) soil C:N:P stoichiometry; AP, accumulated precipitation; AT, accumulated temperature; ΔAET, changes in actual evapotranspiration from 1981–1985 (average) to 2019–2023 (average); T_season_, temperature seasonality; P_season_, precipitation seasonality; C input, N input, and P input, the cumulative C, N, and P inputs over the last 40 years, respectively. The asterisks indicate significance: ^*^ *p* < 0.05, ^**^ *p* < 0.01.

**Figure 6:**
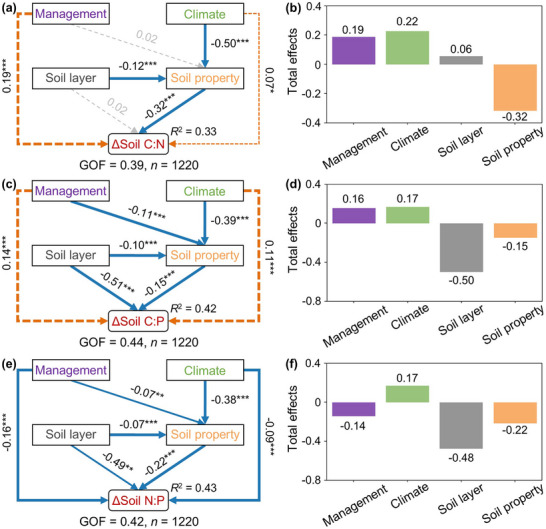
The partial least squares path model (PLS‐PM) depicts factors influencing changes in soil C:N (a), C:P (c), and N:P (e) stoichiometry through direct and indirect pathways. Each subplot shows the total effects of the variables on changes in soil C:N (b), C:P (d), and N:P (f) ratios, respectively. Δsoil C:N, Δsoil C:P, and Δsoil N:P represent the relative change rate, indicating changes in soil C:N, C:P, and N:P in 2023 relative to the 1980s. Orange dashed arrows represent significant positive effects, while blue solid arrows denote significant negative effects (^*^ *p* < 0.05; ^**^ *p* < 0.01; ^***^ *p* < 0.001). Numbers beside the arrows indicate standardized path coefficients. *R* ^2^ reflects the proportion of variance in the dependent variable explained by the model. Gray dashed arrows indicate non‐significant relationships (*p* > 0.05). Management factors include accumulated inputs of carbon, nitrogen, and phosphorus over the past 40 years. Climate factors comprise accumulated temperature and precipitation, variability in actual evapotranspiration, and the seasonality of temperature and precipitation. Soil properties include baseline (1980s) soil C:N:P ratios, pH, sand content, and clay content.

Random forest analysis revealed that the Δsoil C:N:P ratios in each soil layer were mainly influenced by the soil C:N:P_1980s_ ratios and by management (C, N, and P inputs). Specifically, the C:N_1980s_ ratios and C inputs made significant contributions to changes in soil C:N ratios; the C:P_1980s_ ratios and P inputs (P from manure, straw, and mineral fertilizers) contributed significantly to changes in soil C:P ratios; and the N:P_1980s_ ratios, along with N (Similar to P source) and P inputs, significantly affected changes in soil N:P ratios. In addition, soil pH and climatic variables (AT, AP, and P_season_) also had significant effects on changes in soil C:N ratios (**Figure** **5**). However, after controlling for some factors, the AT, AP, P_season_, and soil pH were no longer correlated, in contrast to C, N inputs, and C:N_1980s_, which remained significantly correlated with the Δsoil C:N ratios (Figure S9, Supporting Information). Similarly, the Δsoil C:P and Δsoil N:P ratios were related to management practices (C, N, and P inputs) and baseline soil C:P ratios, regardless of whether climatic factors and soil properties were controlled (Figures S10 and S11, Supporting Information). Partial least squares path modeling was further applied to assess the regulatory pathways through which various factors influence the Δsoil C:N:P ratios (**Figure** **6**). All variables except soil layer exhibited significant direct effects on Δsoil C:N, with soil properties showing the strongest negative effect (−0.32). In addition, both climate and soil layer indirectly regulate changes in soil C:N by affecting soil properties. For Δsoil C:P and Δsoil N:P, the soil layer (−0.51 and −0.49) exerted the strongest negative effects. Management practices positively influenced changes in soil C:N and C:P, but had a negative effect on Δsoil N:P. Although the direct effects of climate on Δsoil C:P and Δsoil N:P were relatively weak, their indirect effects were more pronounced. Overall, management, climate, soil layer, and soil properties jointly regulated changes in soil C:N, C:P, and N:P through both direct and indirect pathways. The random forest models explained 15–48% of the variance, with prediction errors corresponding to ≈0.13–0.20 of the observed range (Table S1, Supporting Information). For the PLS‐PM, the *R* ^2^ values of the endogenous constructs were 0.33–0.43, and the GOF ranged from 0.39 to 0.44. Such levels are typical for large‐scale ecological datasets with high spatial heterogeneity. Therefore, these performance supports the robustness of the identified relative drivers and regulatory pathways, while also indicating that additional unmeasured local factors may contribute to unexplained variability.

## 3 Discussion

This study provided a comprehensive assessment of the temporal dynamics and depth‐dependence of soil C:N:P ratios across a 0–100 cm soil profile in China's croplands, based on repeated measurements of soil C, N, and P at 305 resampling sites in the 1980s and in 2023. Notably, our approach, involving large‐scale soil resampling and laboratory analyses from well‐replicated sites, provides a reliable and rigorous strategy for understanding soil C:N:P dynamics.^[^ ^13^, ^23^, ^24^ ^]^ This research represents the first continental‐scale investigation of these dynamics in agroecosystems, contrasting with conventional methods such as model predictions and meta‐analyses.^[^ ^8^, ^9^, ^10^ ^]^

Over the past 40 years, the C:N and C:P ratios in the entire soil profile of China's croplands increased by 20.18% and 4.49%, respectively, while the N:P ratios decreased by 9.02% (Figure 1). This contrasts with the findings in natural ecosystems.^[^ ^3^, ^25^, ^26^ ^]^ In forest soils, the C:N ratios in the soil profile remained stable, albeit the C:P and N:P ratios increased by 24.82% and 88.41%, respectively, via meta‐analyses over the same period.^[^ ^3^ ^]^ The differences in soil C:N:P ratios between croplands and natural ecosystems can be attributed to both ecosystem types and data acquisition methods. Unlike natural ecosystems, agroecosystems receive additional C inputs (e.g., straw and manure) and nutrient inputs (e.g., chemical fertilizers) to support crop growth. These inputs, characterized by higher C:N and C:P ratios, promote soil C accumulation relative to N and P.^[^ ^19^ ^]^ Additionally, soil N is more susceptible to loss through volatilization and leaching, whereas soil P, despite its low use efficiency, is less mobile and can be immobilized by compounds such as calcium carbonate and metal oxides, reducing its loss and lowering the soil N:P ratios.^[^ ^27^ ^]^ In addition, these changes showed spatial heterogeneity (Figure 2). Variations in soil C:N and C:P ratios decreased with latitude and were influenced by accumulated temperature and precipitation (Figure S2, Supporting Information), suggesting low‐latitude regions are more affected by climate change and human activities.^[^ ^28^ ^]^ Severe native P loss caused high baseline C:P ratios in subtropical and tropical monsoon zones. However, relative changes in C:P ratios were similar across climate zones, indicating long‐term management masked natural differences, leading to anthropogenic homogenization effects.^[^ ^19^ ^]^ N:P ratios had the lowest baseline (3.68) but largest increase (+0.77) in the plateau–alpine zone (Figure 2c). Although warming‐induced freeze–thaw cycles may promote soil P release, excessive N fertilization and atmospheric N deposition caused N to accumulate faster than P.^[^ ^3^ ^]^ In temperate and subtropical zones, declining N:P ratios suggest potential N losses due to precipitation. Overall, 40 years of continuous cropping have exacerbated nutrient imbalances and altered C, N, and P cycling in China's croplands, resulting in the decoupling of these elements under long‐term changing conditions (Figure 3).

Furthermore, the direction of soil C:N:P changes (i.e., increasing or decreasing) was contingent on the soil C:N:P levels in the 1980s (Figure 4). In high C:N soils, microorganisms experience a degree of “N‐starvation”, which induces them to decompose organic matter to obtain N, thus causing a decrease in soil C:N ratios over time.^[^ ^29^, ^30^ ^]^ Conversely, soils with low soil organic C tend to acquire or retain C more readily than soil organic C‐rich soils, while low C:N soils are more likely to increase C storage (and thus soil C:N) when enriched with straw or organic fertilizers.^[^ ^31^ ^]^ Similar patterns were observed for Δsoil C:P and Δsoil N:P ratios. Moreover, the thresholds of Δsoil N:P steadily decreased with soil depth, which underscores the need for vigilance regarding environmental issues associated with N loss. Thus, it is essential to incorporate the responses of deeper soils to long‐term environmental changes into future ecological forecasts, rather than focusing on topsoil alone.

This study further revealed that variations in soil C:N:P ratios were depth‐dependent in croplands (Figures 3 and 4). Across the entire soil profile, the soil C:N ratios increased, while the soil C:P and N:P ratios increased in the topsoil (0–40 cm) but decreased in the subsoil (40–100 cm). These findings not only extended previous research on soil C:N:P changes in the topsoil^[^ ^4^, ^14^, ^25^ ^]^ but also importantly advanced our understanding that elemental stoichiometry may be altered across the entire soil profile in croplands. The reasons for this depth‐dependent pattern may be threefold:

First, the sources and transformations of C, N, and P differ between topsoil and subsoil. The increased C:N and C:P ratios in China's cropland topsoil are primarily due to organic C inputs from practices such as straw returning and organic fertilization (Figure 5). Straw incorporation mainly enriches the plow layer, where residues are retained and decomposed locally, thereby elevating C in the topsoil but contributing little to deeper horizons. Manure and mineral fertilizer application also directly increases topsoil C, N, and P by supplying both organic matter and readily available nutrients, while stimulating microbial activity that accelerates nutrient turnover. Favorable aeration in the topsoil facilitates the conversion of exogenous C to soil organic C by soil microorganisms, leading to a higher C accumulation rate compared to N and P.^[^ ^12^, ^15^ ^]^ This pattern is consistent with observations showing that C accumulation in the topsoil consistently exceeds N gains, resulting in a net increase in topsoil C:N. Likewise, stronger C enrichment relative to P explains the rise in topsoil C:P ratios (Figure S5, Supporting Information). Additionally, the higher use of N fertilizers compared to P fertilizers has resulted in an increased topsoil N:P ratio (Figure S12, Supporting Information). In contrast, subsoil C, N, and P originate from the mobilization of dissolved organic C from the topsoil, leaching of mineral N and P fertilizers, and weathering of subsoil parent materials.^[^ ^32^ ^]^ Low air permeability in the subsoil limits the decomposition of soil C, as substrate quality decreases with soil depth, making organic matter in the subsoil less biodegradable than in the topsoil.^[^ ^33^ ^]^ Conversely, a portion of the soil N may be lost due to organic N being mineralized to available N, followed by crop uptake. The continual downward leaching of N and denitrification lead to an increase in the subsoil C:N ratios.^[^ ^5^, ^34^ ^]^ Although absolute changes in N are negligible or even slightly negative, the relative values appear positive because of the very low baseline concentrations in deeper horizons. Such relative gains, however, do not indicate substantial N storage, as subsoil N remains highly vulnerable to loss. P, in comparison, is consistently retained or slightly enriched through sorption by Fe and Al oxides. This relative pattern, with C increasing more than N but less than P, explains the stoichiometric shifts: continual N loss raises subsoil C:N ratios, whereas stronger P retention compared with both C and N reduces subsoil C:P and N:P ratios. Additionally, the weathering of subsoil parent material provides another important source of soil P beyond soil organic matter degradation and the downward leaching of topsoil P, further reducing subsoil C:P and N:P ratios.^[^ ^4^, ^32^ ^]^ A comprehensive study indicates that soil parent material exerts significant control over P limitation,^[^ ^35^ ^]^ and this is supported by observed differences in subsoil C:P among parent materials (Figure S8, Supporting Information).

Second, climatic changes significantly influence C:N:P ratios in both the topsoil and subsoil (Figure 5), primarily through changes in temperature and precipitation.^[^ ^3^ ^]^ Specifically, warming stimulates crop growth, leading to increased litter‐derived C inputs.^[^ ^36^ ^]^ Additionally, higher temperatures enhance net N mineralization and nitrification, resulting in greater accumulation of C than N in the topsoil and increased C:N ratios.^[^ ^37^ ^]^ Warming also boosts the activities of P‐solubilizing microorganisms, converting more organic P into inorganic forms for crop uptake and subsequent removal from the soil, which might explain the increased topsoil C:P and N:P ratios.^[^ ^32^ ^]^ In deeper horizons, root proliferation under warming conditions provides additional C inputs, while N remains constrained by leaching and denitrification, reinforcing C:N increases.^[^ ^38^, ^39^ ^]^ Precipitation also plays an important role in shaping nutrient dynamics. Increased rainfall promotes greater N leaching, which limits N accumulation relative to C and elevated C:N, while downward P transport combined with strong Fe‐ and Al‐oxide sorption explains the decreases in subsoil C:P and N:P (Figure S5, Supporting Information).^[^ ^5^, ^40^ ^]^ In addition to accumulated temperature and precipitation, precipitation seasonality was significantly related to ΔC:N (Figure 5; Figure S9, Supporting Information). This highlights that the timing and concentration of rainfall events, rather than accumulated precipitation alone, critically influence soil N dynamics. Concentrated precipitation pulses can accelerate N leaching and denitrification, thereby constraining N accumulation relative to C and driving increases in C:N (Figure S5, Supporting Information). These findings suggest that future increases in temperature and precipitation, particularly when combined with more variable rainfall regimes, may intensify N losses relative to C and P, thereby exacerbating soil C, N, and P decoupling in China's croplands.^[^ ^4^ ^]^ Moreover, Δsoil C:N:P exhibited significant differences among climatic zones, particularly in the subsoil rather than the topsoil (Figure S6, Supporting Information). This may be associated with frequent agricultural management practices that weaken the impacts of climate on C:N:P in the topsoil.

Third, the roles of soil structures and minerals in regulating soil C:N:P ratios cannot be overlooked. Agricultural practices, such as the application of manure and incorporation of straw, promote the formation of soil aggregates, which are critical for nutrient fixation and release, as well as for air and water circulation.^[^ ^19^, ^41^, ^42^ ^]^ These aggregates encapsulate soil organic matter and slow the decomposition of soil C, particularly stable humus, thereby increasing topsoil C:N and C:P ratios.^[^ ^43^ ^]^ In the subsoil, abundant clays and oxyhydroxides chemically protect soil C, contributing to higher subsoil C:N ratios.^[^ ^44^ ^]^ Clay minerals, with their high specific surface areas, immobilize P by forming stable chemical bonds through calcium and magnesium bridges.^[^ ^32^, ^45^ ^]^ While this immobilization occurs in the topsoil as well, climatic factors, particularly precipitation, can cause P complexes to leach into the subsoil.^[^ ^32^ ^]^ Additionally, variations in Δsubsoil C:N:P ratios were observed among soil types, with the most significant reductions in C:P and N:P occurring in highly weathered Ultisols and moderately weathered Alfisols (Figure S7, Supporting Information). This is because P in more weathered soils has lower availability and mobility due to its strong binding with iron and/or aluminum oxides released by weathering, making subsoil P more prone to accumulation and lowering C:P and N:P ratios.^[^ ^46^, ^47^ ^]^ Overall, soil C, nutrient sources, climate change, and soil properties collectively drive the depth‐dependent changes in soil C:N:P ratios in Chinese croplands over the last four decades (Figure 6). It should be noted that the random forest and PLS‐PM models provided valuable insights into the drivers of changes in soil C:N:P ratios across cropland profiles. As expected in large‐scale ecological datasets, both models exhibited only moderate explanatory power, which is typical given the high spatial heterogeneity of nationwide studies.^[^ ^48^ ^]^ While both models captured important relationships between key drivers (e.g., climate, management, soil properties), the relatively low explanatory power indicates that some variability remains unexplained. This suggests that local, unmeasured factors, which are difficult to account for at large spatial scales, may play a significant role in soil C:N:P dynamics. Therefore, these models should primarily be interpreted as identifying relative driver importance and regulatory pathways, rather than providing precise site‐level predictions.

It is important to acknowledge that this study may have some limitations. Our dataset includes soil C:N:P data from only two time points (1980s and 2023), despite covering 305 well‐matched cropland sites across China. This approach does not capture the continuous changes in soil C:N:P ratios over the 40 years, leading to an incomplete understanding of these dynamics. Additionally, while we confirmed that land use types, crop types, and cropping systems remained unchanged at the resampling sites over the past 40 years, the absence of historical cultivar data might introduce uncertainties. Different cultivars have varying requirements for soil C, N, and P, as well as different residue inputs, which can influence soil C:N:P ratios. Another methodological limitation arises from both the spatial matching of the 1980s survey sites and the harmonization of soil depths between the two sampling periods. Because the original profiles were recorded using descriptive information rather than precise coordinates, subsequent digitization relied on a place‐name–polygon matching process, and in 2023, we further consulted elderly villagers who had participated in the original survey to refine site identification. Although these efforts minimized relocation bias, some uncertainty in site matching cannot be fully excluded. In addition, in the 1980s, soil samples were collected based on genetic horizons of variable thickness, whereas in 2023, standardized depth intervals (0–20, 20–40, 40–60, and 60–100 cm) were used. To reconcile these differences, we applied regression models (logarithmic, exponential, power, and linear) to interpolate SOC, TN, and TP concentrations at fixed depth intervals. Although the best‐fitting models were selected for each profile based on *R* ^2^ values, this approach introduces model‐based uncertainty, as the true relationship between depth and nutrient concentration may not always conform perfectly to these functional forms. To evaluate this, we conducted a bootstrap sensitivity analysis (Table S2, Supporting Information), which confirmed that the direction of change is statistically robust. Directional stability was generally 80%–90% across layers, and ±2 cm perturbations of horizon boundaries had negligible effects, indicating that uncertainty in depth matching exerts little influence on the conclusions. Despite these limitations, the uncertainties are mitigated by our large‐scale resampling survey, which involved rigorous and effective efforts. The soil organic C, total N, and total P used to calculate Δsoil C:N:P ratios were derived from actual laboratory measurements, not simulated values, representing a significant advancement in the investigation of soil C:N:P ratios in croplands at this scale.

## 4 Conclusion

To summarize, this study offers essential insights into the spatiotemporal dynamics of soil stoichiometry in croplands. Over the last four decades, the soil C:N and C:P ratios have increased, while N:P ratios have decreased in China's croplands. These changes were depth‐dependent, with contrasting patterns in the topsoil and subsoil, and were primarily driven by ongoing agricultural practices. The initial soil C:N:P_1980s_ influenced the direction of these changes and provides benchmarks for future agronomic management. In addition, given that China represents one of the largest and most intensively managed agricultural systems globally, the findings from our study may also serve as a valuable reference for other countries undergoing similar trajectories of land‐use intensification. The depth and long‐term patterns of soil stoichiometric changes revealed here can help inform nutrient management strategies and modeling efforts in other agricultural countries, especially those facing the dual challenges of maintaining productivity and ensuring long‐term soil sustainability.

## 5 Experimental Section

### Study Region

This study focused on the extensive agricultural soils of China, spanning ≈25° of latitude (from 18.73°N to 48.02°N) and 55° of longitude (from 76.45°E to 131.59°E), and encompassing an extensive range of climates and soil types. The mean annual temperature (MAT) in the region ranges from −7.18 to 23.95 °C, while the mean annual precipitation (MAP) varies from 73.36 to 2571.84 mm. Given this significant climatic diversity, the region was divided into five major climatic zones (temperate continental, temperate monsoon, subtropical monsoon, tropical monsoon, and plateau‐alpine climatic zone) (Figure S13, Supporting Information).^[^ ^49^ ^]^ Nearly all major soil types found in China were included in this investigation (e.g., red, brown, and black soils). For international comparison, the Chinese soil taxonomy was aligned with the United States Department of Agriculture (USDA) soil taxonomy. The main identified soil orders were Alfisols, Aridisols, Entisols, Inceptisols, Mollisols, Ultisols, and Vertisols.^[^ ^50^ ^]^ Meanwhile, parent materials were classified into four primary rock categories (sedimentary, plutonic, volcanic, and metamorphic).^[^ ^51^ ^]^ The major crops grown in the study region included wheat, maize, and rice. Given the variability in climatic conditions, as well as soil types combined with diverse agricultural activities, this region provides an ideal setting for studying how soil C and nutrient dynamics differ under varying environmental conditions and agricultural practices.

To assess the status of soil resources, fertility, and production potential across Chinese croplands, the Second Soil Inventory was conducted in the 1980s (Figure 1). The primary findings were published in the China Soil Series volumes I–VI (National Soil Survey Office, 1993–1996). These volumes provided descriptions of typical soil profiles from croplands across the country, including details on soil series, location, and land‐use, along with some analytical data, such as soil organic C (SOC), total N (TN), total P (TP) concentrations, and other key soil physicochemical properties. These parameters provided critical baseline data for analyzing patterns in soil C:N:P ratios across different regions, which enable in‐depth assessments of spatial variations, driving factors, and long‐term changes influenced by natural conditions and anthropogenic activities. Due to the variable thickness of soil genetic horizons, the relationships between soil depth and the concentrations of SOC was fitted, TN, and TP for each profile using logarithmic, exponential, power, and linear regression models. The best‐fitting model, determined by the highest coefficient of determination (*R* ^2^), was then selected to estimate SOC, TN, and TP concentrations at standardized depth intervals (0–20, 20–40, 40–60, and 60–100 cm), facilitating comparisons between the two time periods (Table S3, Supporting Information). For each profile, it was assumed that the measured value of a given horizon represents the concentration at the midpoint of that horizon, and the predicted value at the midpoint of each fixed depth interval was used to represent the average concentration within that interval.^[^ ^52^ ^]^ To quantify the uncertainty introduced by converting soil genetic horizons (1980s) to fixed‐depth intervals (2023), a residual bootstrap (B = 1000) procedure was performed within each profile. Residuals from the depth–concentration regressions were resampled, refitted, and propagated to estimate layer means at 0–20, 20–40, 40–60, and 60–100 cm. For each bootstrap replicate, site‐level relative changes (%) between 2023 and the 1980s were calculated and then averaged across sites. The mean relative change with bootstrap 95% confidence intervals (Mean [95% CI] %) were reported, the proportion of replicates consistent in sign (Directional stability, %), and the sensitivity of results to ±2 cm boundary perturbations (Boundary Δmean, Boundary Δmean/halfCI, and qualitative impact flag) (Table S2, Supporting Information).

### Resampling Campaign

To quantify Δsoil C:N:P ratios across China's croplands over the past 40 years, a broad‐scale resampling campaign was conducted in 2023, which built upon the historical soil sampling sites from the Second Soil Inventory. During the second national soil survey in China, soil profile locations were recorded based on descriptive information such as village names and local landscape features (e.g., “eastern slope of the village”), rather than precise geographic coordinates. In subsequent national digitization efforts, these legacy sites were georeferenced through a place–name–polygon matching process, in which each soil profile was assigned to the centroid of a corresponding soil mapping unit based on the available site descriptions. The coordinates provided in all accessible versions of the Second National Soil Survey dataset were retrospectively assigned using this method and have since served as the standard spatial reference in subsequent studies. In the 2023 resampling campaign, GPS was used to locate these coordinates, and elderly villagers who had participated in the original 1980s survey were further consulted to refine site identification. To avoid bias from differing sampling techniques, the soil samples were collected according to the same protocols. Specifically, three 10 × 10 m sample plots were established around the original coordinates. In each plot, seven replicate soil profiles were dug following an “S” pattern. Soil samples were collected from four distinct depths (0–20, 20–40, 40–60, and 60–100 cm) in each soil profile. The seven samples from each soil layer were mixed to create a composite sample. In total, 12 soil samples (three replicate plots × four soil layers) were collected from each site. In addition, a steel cylinder with a fixed volume of 100 cm^3^ was inserted into the soil profile, with its center aligned to the midpoint of each target soil layer (i.e., at depths of 10, 30, 50, and 80 cm), to collect soil samples for BD determination at each depth. Despite practical constraints (urbanization, road reconstruction, and the “grain for green” program), 305 matched resampling sites were successfully obtained (Figure 1). These resampled sites represented the original sampling locations across all Chinese provinces, except for Tibet, where land‐use changes prevented resampling. The inclusion of these resampled sites provided a representative overview of the geographical scope of the original study. In total, 6405 soil profiles were excavated and derived 3660 composite soil samples. Critically, reliable evidence from questionnaire surveys and the China County Statistical Yearbook (https://www.stats.gov.cn/sj/ndsj/) confirmed that staple crops (maize, wheat, and rice) had been continuously grown at these sampling sites over the past 40 years, and that the dominant cropping systems (crop identity and single versus double cropping) remained consistent at these sites. This continuity in land use ensured the validity of the resampling efforts, which provided robust data to assess long‐term changes in soil C:N:P ratios and their driving factors, particularly under consistent agricultural practices.

### Laboratory Measurements

To ensure consistency with the original 1980s dataset, identical techniques were applied for the determination of soil variables in the resampled dataset. Specifically, after air‐drying the soil samples, roots and stones were meticulously removed. The remaining soil was then ground into a fine powder and sifted through a 2 mm sieve to ensure uniformity and to remove any remaining debris. The soil organic C concentrations were measured using the potassium dichromate (K_2_CrO_7_) oxidation‐ferrous sulphate (FeSO_4_) titration method. The principle behind this method is that soil organic C is oxidized by K_2_CrO_7_, which produces CO_2_ at ≈200 °C. The remaining K_2_CrO_7_ was titrated with FeSO_4,_ and the soil organic C concentration was calculated based on the quantity of K_2_CrO_7_ consumed.^[^ ^53^ ^]^ The total N concentration was measured using the Kjeldahl method, which involved high‐temperature digestion to convert N to ammonium (NH_4_^+^). The NH_4_^+^ in the digested solution was released through distillation, absorbed by boric acid, and finally titrated with a diluted acid standard solution to quantify the total N concentration.^[^ ^54^ ^]^ For the total P concentration, the molybdate colorimetric test was employed following perchloric acid (HClO_4_) digestion.^[^ ^55^ ^]^ Briefly, the soil P was digested into orthophosphate, which formed blue complexes upon reaction with ammonium molybdate. The total P concentration was calculated based on the absorbance value measured by a spectrophotometer. Traditionally, the fixed depth (FD) was selected to explore the variations in soil resources over time. However, soil BD can vary across soil profiles over time. FD‐based comparisons can introduce significant uncertainties. To mitigate this issue, the equivalent soil mass (ESM) method was used as an alternative to FD with no adverse effects from changes in the bulk density.^[^ ^56^ ^]^ This approach eliminated the adverse effects of bulk density fluctuations, thereby ensuring more accurate assessments of long‐term changes in soil C:N:P. The following equations were used to calculate the relative changes in soil C:N:P ratios over the last 40 years:

(1) $$\Delta \mathrm{soil}\;C:N=\mathrm{soil}\;C:N_{2023}-\mathrm{soil}\;C:N_{1980}$$

(2) $$\Delta \mathrm{soil}\;C:P=\mathrm{soil}\;C:P_{2023}-\mathrm{soil}\;C:P_{1980}$$

(3) $$\Delta \mathrm{soil}\;N:P=\mathrm{soil}\;N:P_{2023}-\mathrm{soil}\;N:P_{1980}$$

where, soil C:N, C:P, and N:P are converted to molar ratios.

The pH of each soil sample was measured using a pH meter in a 1:2.5 mixture of soil and deionized water. The mixture was shaken for 30 min and then allowed to settle for 5 min before obtaining the measurement.^[^ ^57^ ^]^ The sedimentation technique was employed to determine the soil texture (sand, clay, and silt contents).^[^ ^58^ ^]^ In brief, the soil samples were chemically treated to disperse the particles in water. A hydrometer was used to measure changes in the density of the suspension at specific time intervals, which allowed for the calculation of the proportions of particles of different sizes. Soil BD was determined by drying the soil samples at 105 °C to a constant weight and calculating the ratio of the oven‐dried mass to the volume of the steel cylinder (100 cm^3^).^[^ ^13^ ^]^ Soil CEC was determined by ammonium acetate exchange (pH 7.0).^[^ ^59^ ^]^ Briefly, air‐dried soil was saturated with 1 m NH_4_OAc, washed with ethanol to remove excess salts, and NH_4_^+^ was displaced with KCl solution. Extracted NH_4_^+^ was quantified (distillation or colorimetry), yielding CEC as cmol kg^−1^ dry soil.

### Other Datasets

In addition to soil properties, climate and management practices played crucial roles in influencing soil C:N:P ratios. To account for the cumulative climatic effects on the soil C:N:P over the last 40 years, temperature and precipitation data was obtained for the original sampling sites from 1980s to 2023 using the ERA5‐Land dataset, which was provided by the European Center for Medium‐Range Weather Forecasts (https://cds.climate.copernicus.eu/). The raw data consisted of monthly mean temperature and precipitation rasters. From these, MAT and MAP for each site was derived. The accumulated temperature (AT) and precipitation (AP) were further calculated to quantify the long‐term climatic impacts. The process was achieved using the following equations:

(4) $$\mathrm{MAT}i\left(x,y\right)=\frac{1}{12}\sum_{m=1}^{12}Ti,m(x,y)$$

(5) $$\mathrm{MAP}i\left(x,y\right)=\frac{1}{12}\sum_{m=1}^{12}Pi,m(x,y)$$

(6) $$\mathrm{AT}\left(x,y\right)=\sum_{i=1980}^{2023}\mathrm{MAT}i(x,y)$$

(7) $$\mathrm{AP}\left(x,y\right)=\sum_{i=1980}^{2023}\mathrm{MAT}i(x,y)$$

where *i* is the year; (*x,y*) is a pixel in the raster, i.e., the latitude and longitude of the sampling site; and *m* is the month within a given year.

To account for the seasonal variability of climate, data on temperature and precipitation seasonality were obtained from WorldClim (https://worldclim.org/). In addition, actual evapotranspiration (AET), which is considered a key factor influencing global system dynamics, was obtained as annual time‐series data from the Global Resources Data Cloud (www.gis5g.com).

Management practices and grain yield data were recorded by consulting the China County Statistical Yearbook and questionnaire surveys, in which management practices focused on C, N, and P inputs. The cumulative C, N, and P inputs were obtained by first estimating annual inputs from different sources (straw incorporation, crop residues, manure, and mineral fertilizers) and then summing these over the past four decades.

The annual C inputs were primarily derived from the incorporation of straw (*C*_straw_), crop residues (*C*_residues_), and manure (*C*_manure_), and calculated using the following equation:

(8) $$\mathrm{Annual}\;C\;\mathrm{input}=C_{\mathrm{straw}}+C_{\mathrm{residues}}+C_{\mathrm{manure}}$$

The national mean moisture content (14%) and C concentrations (399, 444, and 418 g kg^−1^) were utilized from straw of wheat, maize, and rice to calculate the C inputs:

(9) $$C_{\mathrm{straw}}=Y_{\mathrm{straw}}\times \left(1-14\%\right)\times C_{S}/1000$$

where *Y*_straw_ and *C*_s_ are the crop straw yield (kg ha^−1^) and C concentration (g kg^−1^), respectively.

Crop residue C inputs included roots and root stubble C inputs. C inputs from roots were estimated based on the belowground to aboveground biomass ratios. Particularly, the ratios of root biomass to total crop biomass were set as 30% for wheat, 26% for maize, and 30% of aboveground biomass for rice.^[^ ^60^ ^]^ According to the literature, 75.3% and 85.1% of the total root biomass of wheat and maize, respectively, resided within the top 20 cm of the surface soil. The contributions of root stubble to C inputs were assessed using the ratios of stubble to straw biomass, which were 15% for wheat, 3% for maize, and 5.6% for rice.^[^ ^61^ ^]^ The total C inputs were calculated using the following formula:

(10) $$C_{\mathrm{residues}}=\left(\left(Y_{\mathrm{grain}}+Y_{\mathrm{straw}}\right)\times R_{r}\times R_{b}+Y_{\mathrm{straw}}\times S_{r}\right)\times \left(1-14\%\right)\times C_{s}$$

where *Y*_grain_ and *Y*_straw_ are grain and straw yields (kg ha−^1^), respectively; *R*_r_ is the root to total plant biomass ratios (root‐crown ratios); *R*_b_ is the percentage of root biomass located in the topsoil (e.g., within the top 20 cm); *S*_r_ is the stubble to straw biomass ratios; and *C*_s_ is the *C* concentration of straw (g kg^−1^).

For manure inputs, site‐level questionnaire surveys were conducted for each resampling field. These surveys recorded manure type (pig, cattle, sheep, etc.), years of application, annual application rates, and whether the reported values were given in fresh or dry weight. These amounts were converted to elemental C, N, and P inputs using the mean nutrient concentrations and moisture contents reported in the China Organic Fertilizer Nutrient Records. C inputs from manure were calculated using the following equation:

(11) $$C_{\mathrm{manure}}=C_{m}\times \left(1-W\%\right)\times \mathrm{Weight}/1000$$

where *C*_m_, W%, and Weight are the organic *C* concentration (g kg−^1^), water content, and fresh weight of applied manure (kg ha−^1^), respectively.

The annual *N* and *P* inputs aside from straw incorporation, crop residues, and manure that should also be focused on include *N* (*N*_mineral_) and *P* (*P*_mineral_) inputs from mineral fertilizers:

(12) $$\mathrm{Annual}\;N\;\mathrm{input}=N_{\mathrm{straw}}+N_{\mathrm{residues}}+N_{\mathrm{manure}}+N_{\mathrm{mineral}}$$

(13) $$\mathrm{Annual}\;P\;\mathrm{input}=P_{\mathrm{straw}}+P_{\mathrm{residues}}+P_{\mathrm{manure}}+P_{\mathrm{mineral}}$$

It should be noted that the *N* and *P* inputs from mineral fertilizers (*N*_mineral_ and *P*_mineral_), obtained from the China County Statistical Yearbook, were converted to pure *N* or *P* concentrations. If only the total mineral fertilizer application were available, the quantities of *N* or *P* fertilizer inputs for each year were estimated based on the *N* or *P* ratios of the total mineral fertilizer application over the past 40 years. Similarly, the formulas used to calculate N and P inputs from the incorporation of straw, crop residues, and manure followed the same structure as those employed for the C inputs. The only difference was that the values corresponding to the C concentrations in the formulas were replaced with those of the N and P concentrations. The annual inputs of C, N, and P from straw, residues, manure, and mineral fertilizers (derived from Equations 8, 12, and 13) were then summed across the study period (over the past four decades) to obtain cumulative inputs.

### Statistical Analyses

Statistical data analyses were performed according to the following four steps. First, to ensure clarity, we standardized the terminology used for describing changes in soil variables. Absolute change was defined as the direct difference between values measured in 2023 and those in the 1980s, expressed in the same units as the variable (e.g., g kg^−1^ for SOC, TN, and TP). Relative change was defined as the proportional difference relative to the 1980s baseline, calculated as (*X*_2023_−*X*_1980s_)/*X*_1980s_, and expressed as a percentage. Accordingly, all Δ‐variables (e.g., ΔC:N, ΔC:P, ΔN:P, ΔAET) denote relative changes (%), whereas differences in SOC, TN, and TP concentrations reported in units (g kg^−1^) represent absolute changes. A summary table (Table 1) provides an overview of all key variables, abbreviations, and units used in this study. ΔC:N:P values were tested for normality, and one‐way analysis of variance (ANOVA) was used to detect differences in Δsoil C:N:P among profiles, as well as variations among climatic zones, soil types, and parent material types within the same profile. Before the multifactor analysis, multicollinearity among predictor variables was assessed using the variance inflation factor (VIF), with a threshold of 5 to ensure model stability. All variables with VIF values above this threshold were removed (Tables S4–S6, Supporting Information).^[^ ^62^ ^]^

Second, random forest analysis was conducted using the “randomForest” and “rfPermute” packages in R to identify the most important predictors of ΔC:N:P. Random forest is a non‐parametric ensemble learning algorithm that builds multiple regression trees through bootstrap sampling and random feature selection, allowing for robust modeling of nonlinear relationships and variable interactions while minimizing the risk of overfitting. Each model consisted of 500 trees grown to full depth using default settings. Variable importance was assessed using two complementary approaches. First, the mean decrease in impurity (MDI) was calculated based on the frequency and effectiveness of a variable in reducing node variance across all trees. Second, permutation importance was determined by randomly shuffling the values of each predictor and measuring the resulting increase in out‐of‐bag (OOB) mean squared error (MSE) between observations and predictions. A greater MSE increase indicated higher predictor importance. To evaluate statistical significance, 100 permutations were performed using the “rfPermute” package. Importance values were normalized to allow for cross‐variable comparison, and predictors with *p*‐values below 0.05 were considered significant. The final output provided an assessment of the relative contributions of Management (C, N, and P inputs), Climate (accumulated temperature and precipitation, AET variability, and temperature and precipitation seasonality), and Soil properties (soil pH, sand, clay, and baseline (1980s) soil stoichiometry) to changes in soil stoichiometry. Model performance was further evaluated using the OOB *R* ^2^ (% Var explained) and the root mean square error (RMSE), where RMSE is the square root of the MSE. For interpretability, RMSE was further normalized relative to the observed range (NRMSE) and to the standard deviation (CV(RMSE)), providing complementary assessments of error magnitude with respect to the total spread and inherent variability of the data.

Third, to further investigate the drivers of Δsoil C:N:P identified by the random forest analysis, partial correlation analysis was employed to isolate the independent effects of each factor while controlling for the influence of others. Initially, the confounding variables were utilized as independent variables to perform regression analysis on target variables, to calculate the relationships between each variable and the confounding variables. Subsequently, the influences of the confounding variables were removed through the regression model to yield the residuals of the target variable. Finally, partial correlation analysis assessed the pure association after controlling for the confounding variables by calculating the correlations between these residuals. This method effectively excluded the interference of confounding factors, allowing a more accurate assessment of the independent relationships among the main variables. By controlling for confounding variables, this analysis computed the partial correlation coefficients between each independent variable and soil ΔC:N:P to reflect the net correlation after eliminating the influences of other variables. These coefficients, ranging from −1 to 1, represented the net correlation once the influences of other variables were eliminated. Statistical significance was evaluated through *p*‐values. This approach enables the control of one or more variables and ensures that the findings solely reflect the direct correlations between the variables of interest. A higher absolute value of the coefficient suggests a more substantial independent impact of a given variable on soil ΔC:N:P.

Fourth, to disentangle the direct and indirect effects of multiple drivers on soil stoichiometry changes, partial least squares path modeling (PLS‐PM) was applied using the “plspm” package in R. This method was well‐suited for ecological datasets with complex multicollinearity and latent constructs. Four latent variables were defined: Management (C, N, and P inputs), Climate (accumulated temperature and precipitation, AET variability, and temperature and precipitation seasonality), Soil properties (soil pH, sand, clay, and baseline (1980s) soil stoichiometry), Layer (soil depth layer), and the response variable Changes in soil stoichiometry. The structural model was constructed based on hypothesized directional relationships among these latent constructs. All manifest variables were modeled in mode A (reflective). Model performance was evaluated by examining path coefficients, *R* ^2^ values, and the goodness‐of‐fit (GOF) index.

## Conflict of Interest

The authors declare no conflict of interest.

## Author Contributions

The original concepts were conceived by A.C. and M.X. Sampling collection and soil characterization were carried out by A.C., X.S., and Z.Z. Data analyses were done by X.S. The manuscript was prepared by X.S. and A.C. All authors contributed intellectual input and assistance to this study.

## Supporting information



Supporting Information

## Data Availability

The data and code used in this study are available at https://doi.org/10.6084/m9.figshare.30153562.
