# Supplementary material for: Depth‐Dependence Changes in Soil Stoichiometry in China's Croplands Over the Past Four Decades
Source: Adv Sci (Weinh). 2025 Sep 25;12(47):e06489. doi: 10.1002/advs.202506489 (PMC12713096; doi:10.1002/advs.202506489)
Supplement: Supplementary file 1 — Supporting Information [file ADVS-12-e06489-s001.docx]

**Supporting information for**

**Depth-Dependence Changes in Soil Stoichiometry in China’s Croplands over the Past Four Decades**

Xiaodong Sun^a, b^, Zhenghu Zhou^c^, Yiqi Luo^d^, Qingzhu Gao^a^, Hu Li^e^, Shuo Liu^a^, Minggang Xu^b*^, Yu’e Li^a*^, Andong Cai^a*^

*^a^Institute of Environment and Sustainable Development in Agriculture, Chinese Academy of Agricultural Sciences, Beijing, 100081, China*

*^b^Soil Health Laboratory of Shanxi Province, Institute of Eco-environment and Industrial Technology, Shanxi Agricultural University, Taiyuan, 030031, China*

*^c^Center for Ecological Research, Key Laboratory of Sustainable Forest Ecosystem Management, Ministry of Education, Northeast Forestry University, 26 Hexing Road, Harbin 150040, China*

*^d^School of Integrative Plant Science, Cornell University, Ithaca, NY, USA*

*^e^Institute of Agricultural Resources and Regional Planning, Chinese Academy of Agricultural Sciences, Beijing 100081, China*

*Corresponding authors: Tel.: +86-10-82106022, Fax: +86-10-82106022, E-mail: [caiandong@caas.cn](mailto:caiandong@caas.cn), liyue@caas.cn, and [xuminggang@caas.cn](mailto:xuminggang@caas.cn)

**This file includes,**

**Figures S1 to S13**

**Tables S1 to S6**


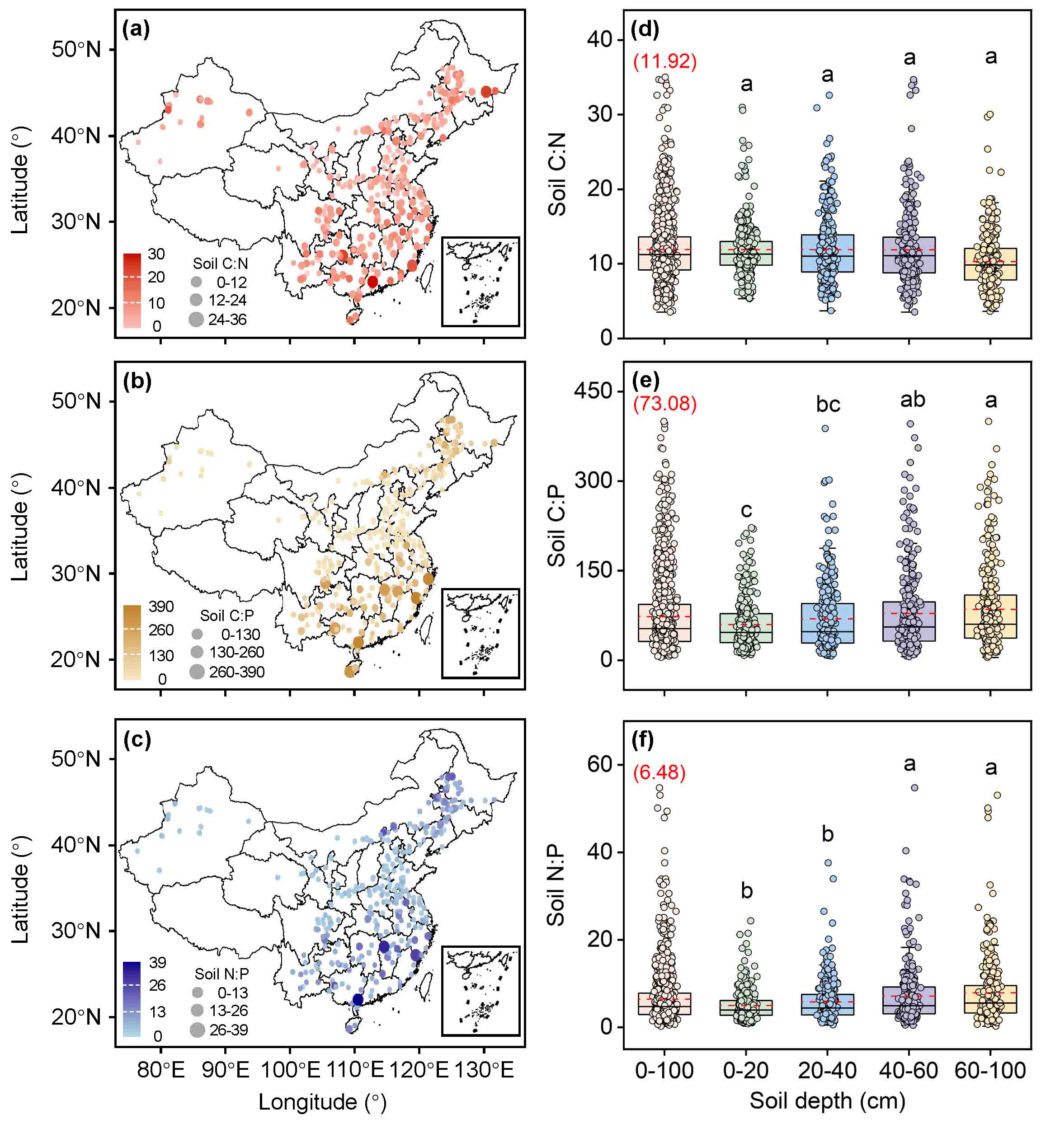


**Figure S1. The initial (1980s) soil stoichiometry status in China’s cropland.** **a-c,** distribution of whole profile soil C:N **(a)**, C:P **(b)**, and N:P **(c)** in 1980. **d-f,** different profile soil C:N **(d)**, C:P **(e)**, and N:P **(d)**. Central line and whiskers (red) in each box represent the median and mean, respectively. Boxes indicate the interquartile range between 25th and 75th percentile. The significant differences between soil depth are represented by different lowercase letters, *p* < 0.05.


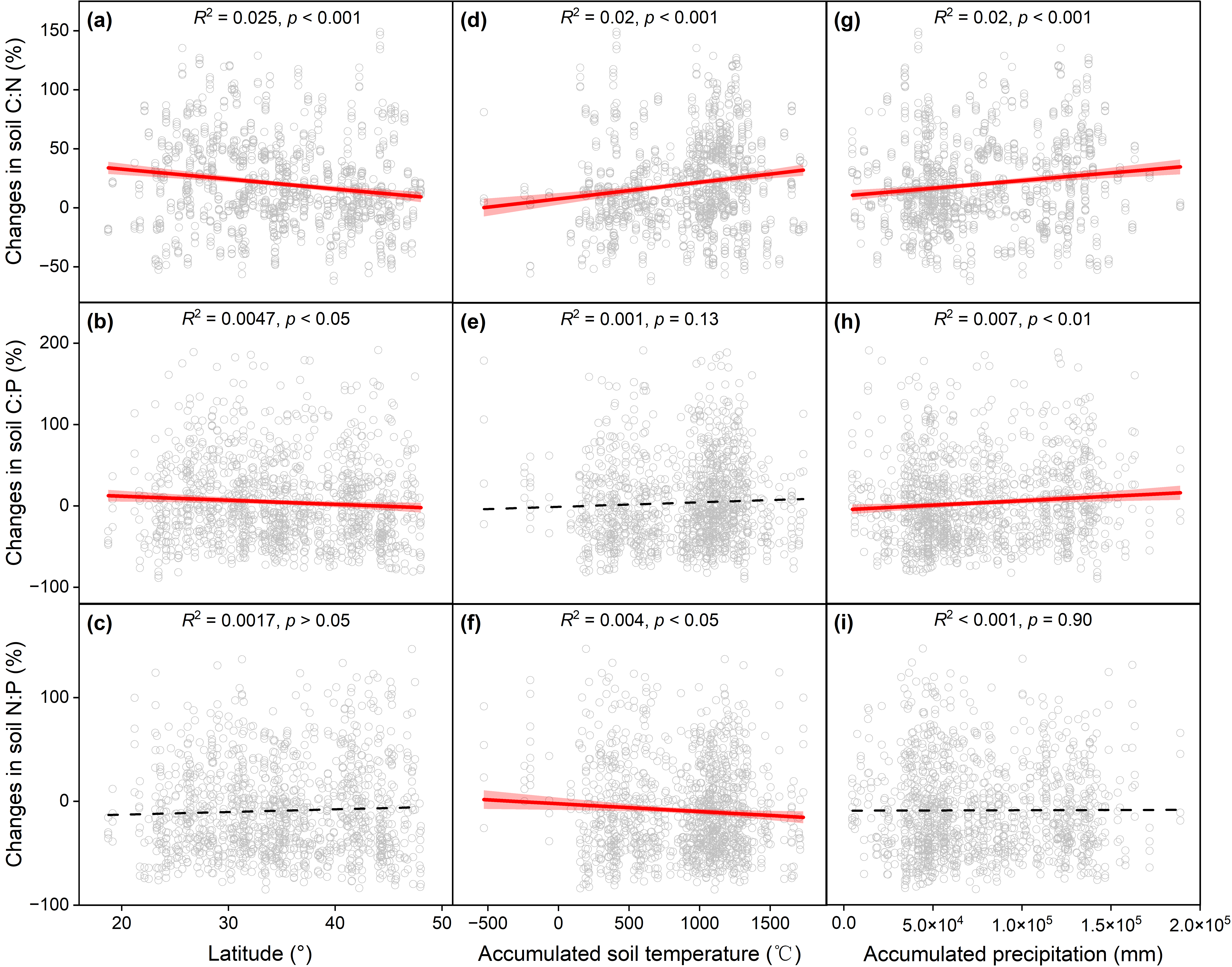


**Figure S2. Relationship between changes in soil C:N, C:P, and N:P ratios and latitude (a-c), accumulated temperature (d-f), and precipitation (g-i).** The solid line represents the significant linear regression (*p* < 0.05), and the shading indicates the 95% confidence intervals.


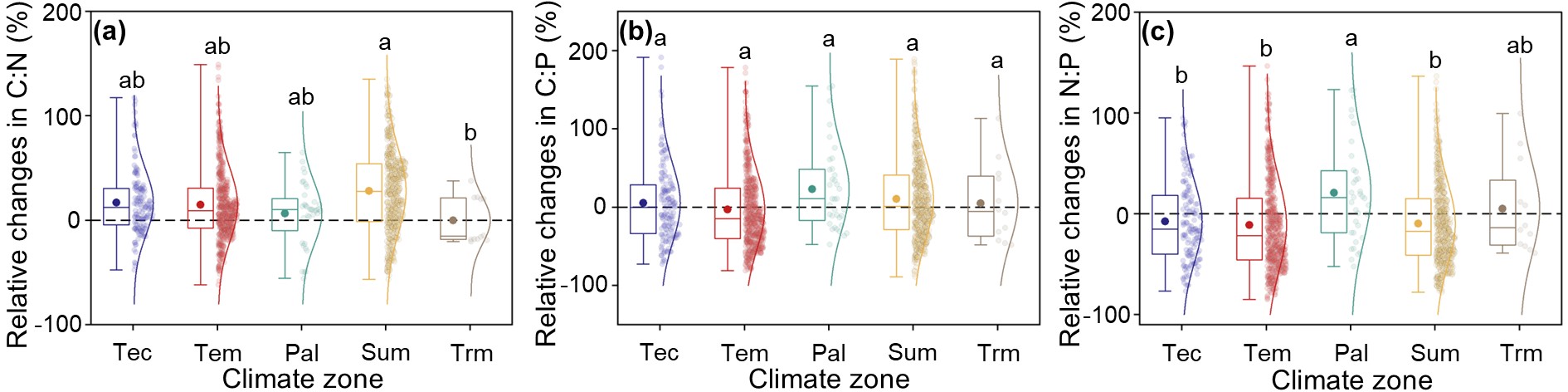


**Figure S3. Relative changes in soil C:N (a), C:P (b), and N:P (c) ratios across different climate zones in China’s croplands.** Tec, temperate continental zone; Tem, temperate monsoon zone; Pal, Plateau-alpine zone; Sum, subtropical monsoon zone; Trm, tropical monsoon zone. Box spans the 25th–75th percentiles, solid line indicates the median, filled dot in box denotes the mean, whiskers extend to the minimum and maximum. The significant differences between climatic zones are represented by different lowercase letters, *p* < 0.05.


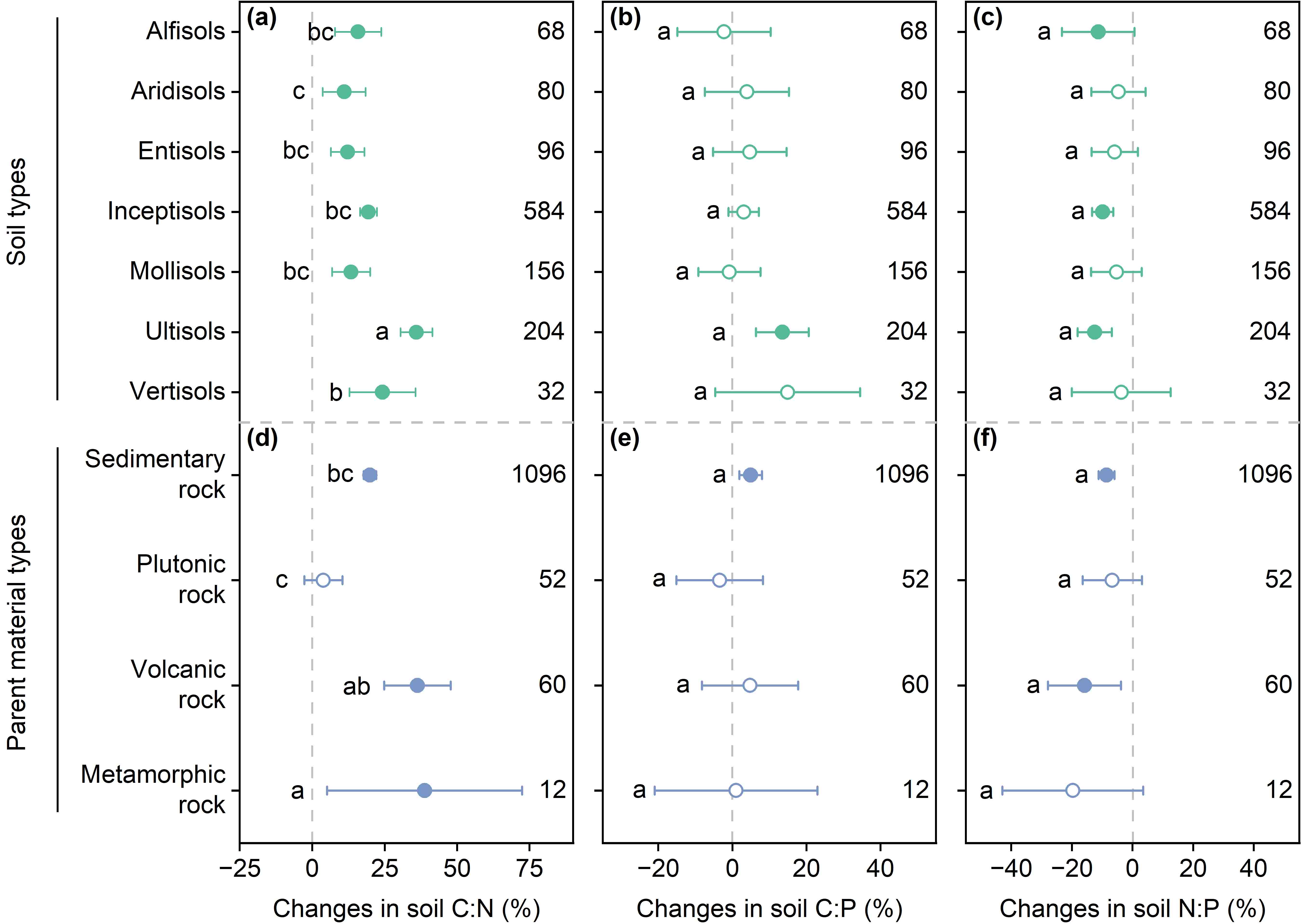


**Figure S4. Changes in soil C:N:P across whole soil profiles in different** **soil types (a-c), and parent material types (d-f).** Tem, temperate monsoon zone; Pal, Plateau-alpine zone; Sum, subtropical monsoon zone; Trm, tropical monsoon zone. The numbers at right y-axes represent the sample size. Error bars represent 95% confidence intervals. The closed and open symbols indicate significant and non-significant effects, respectively. Different letters mean significant differences between different soil types and parent material types, *p* < 0.05.


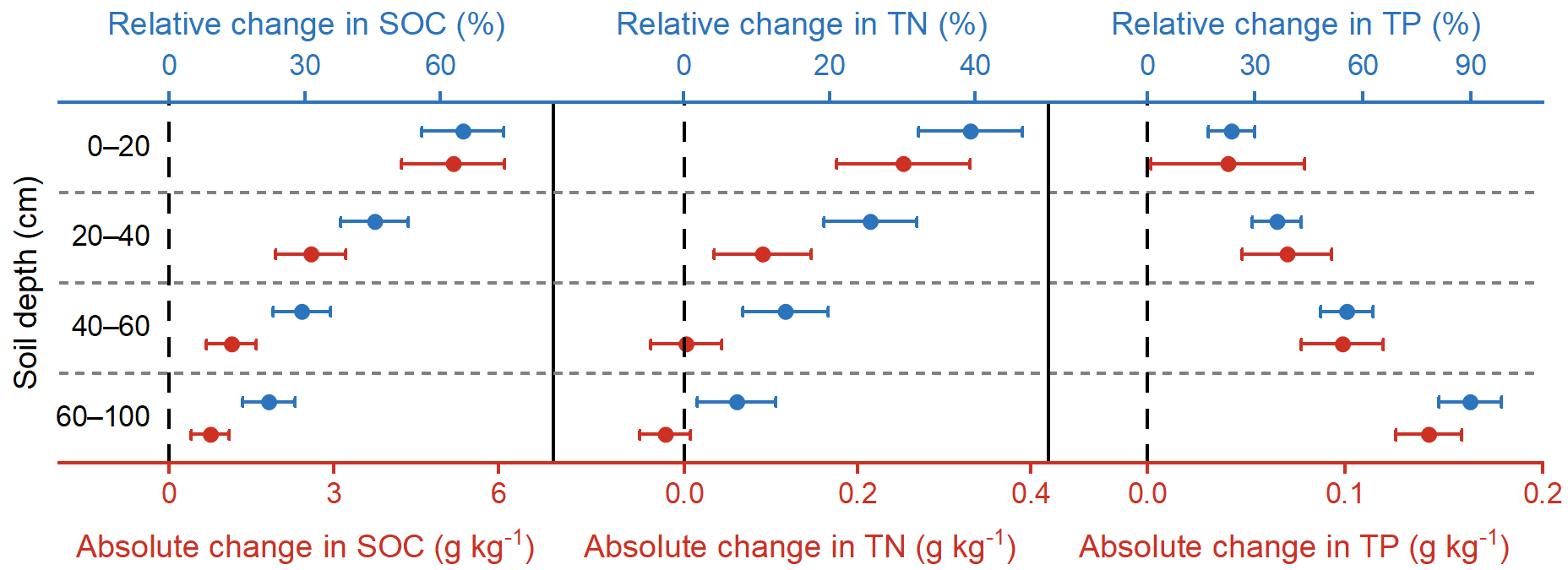


**Figure S5. Relative and absolute changes in soil organic carbon (SOC), total nitrogen (TN), and total phosphorus (TP) across soil depths.**


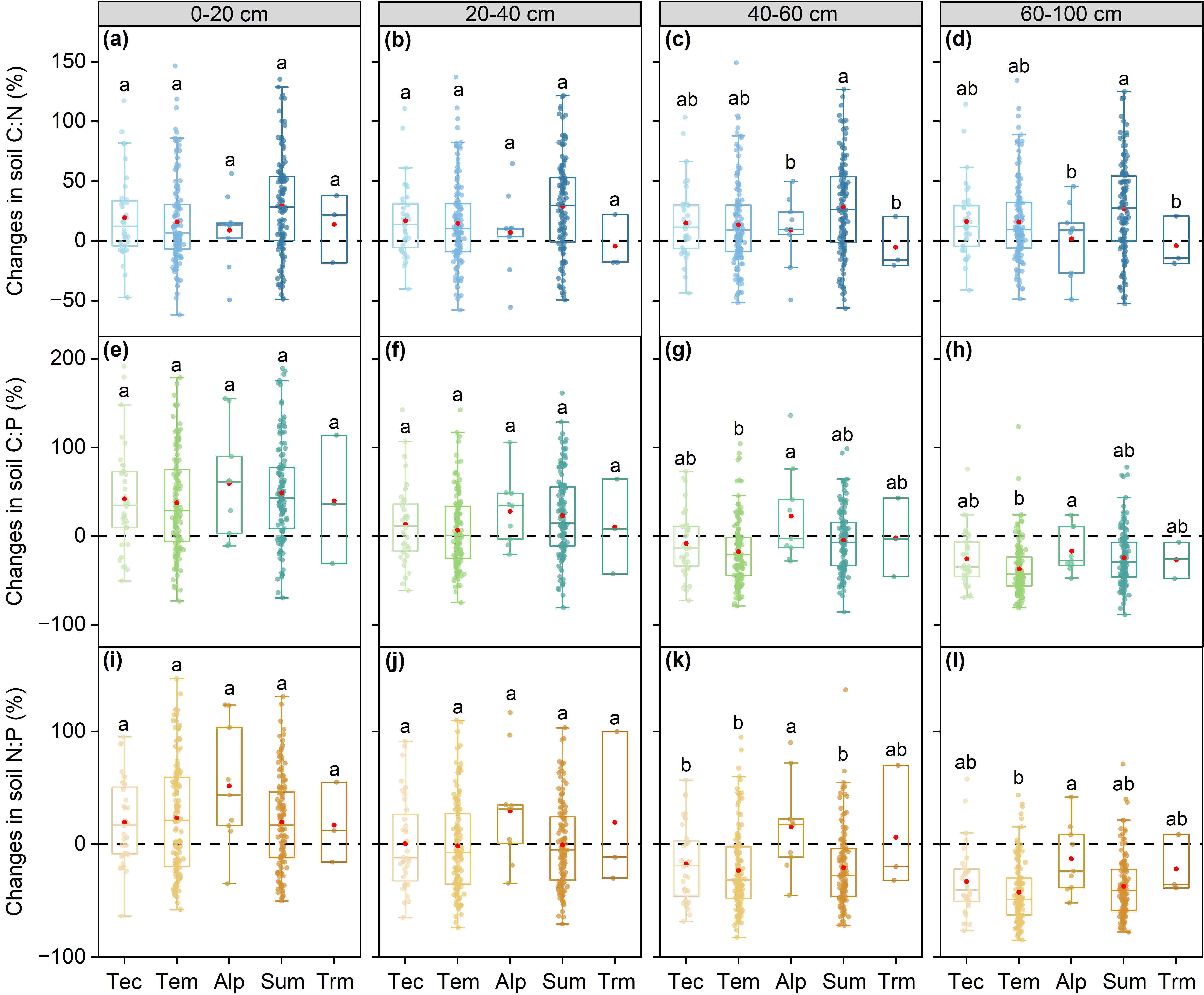


**Figure S6. Changes in soil C:N:P stoichiometry of China’s cropland across soil profiles in different climatic zones over the past 40 years.** **a-d,** changes in soil C:N at 0-20 **(a)**, 20-40 **(b)**, 40-60 **(c)**, and 60-100 cm **(d)** in different climatic zones. **e-h,** changes in soil C:P at 0-20 **(e)**, 20-40 **(f)**, 40-60 **(g)**, and 60-100 cm **(h)** in different climatic zones. **i-l,** changes in soil N:P at 0-20 **(i)**, 20-40 **(j)**, 40-60 **(k)**, and 60-100 cm **(l)** in different climatic zones. Tec, temperate continental zone; Tem, temperate monsoon zone; Pal, Plateau-alpine zone; Sum, subtropical monsoon zone; Trm, tropical monsoon zone. Box spans the 25th–75th percentiles, solid line indicates the median, filled dot in box denotes the mean. The significant differences between climatic zones are represented by different lowercase letters, *p* < 0.05.


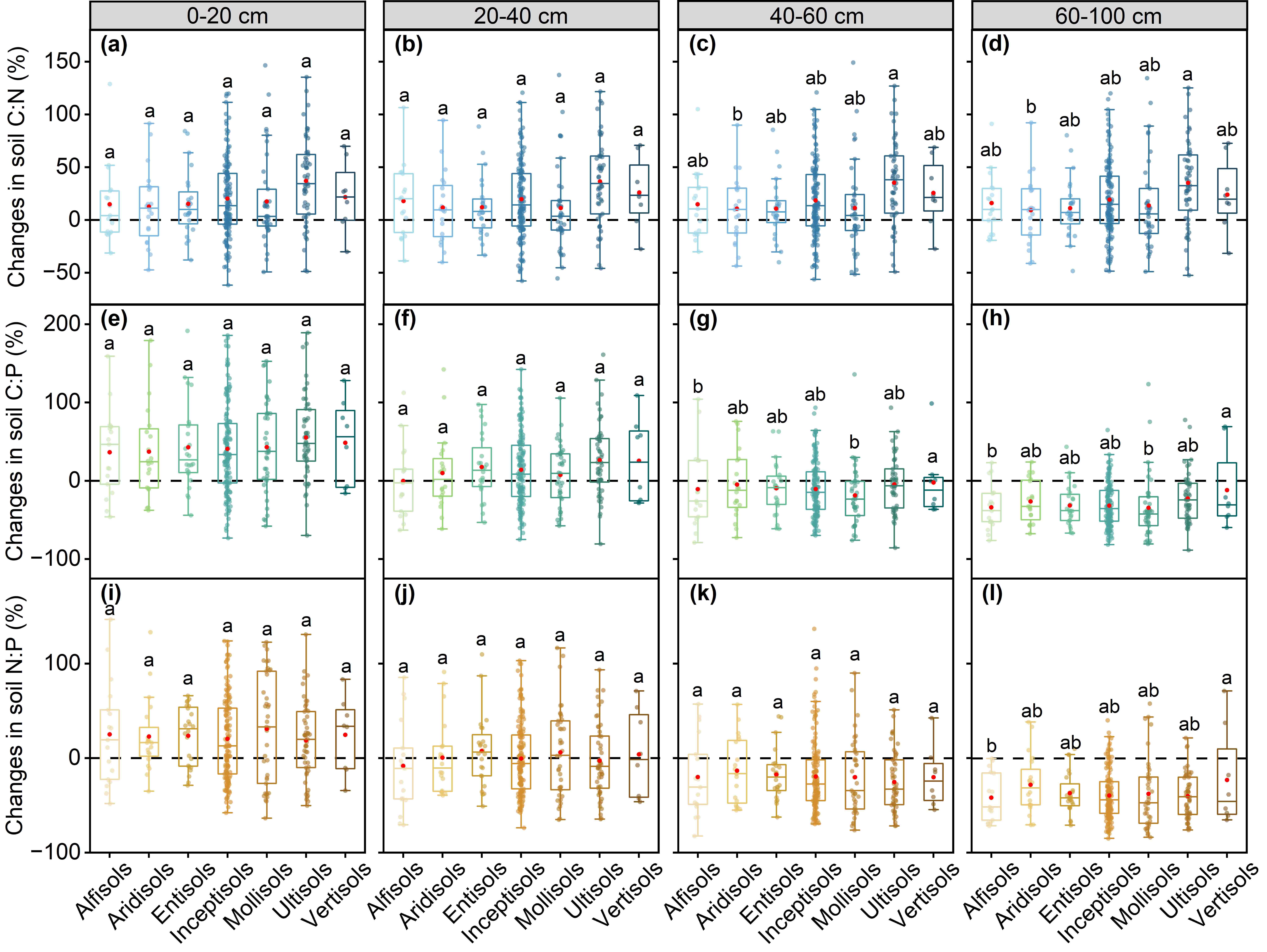


**Figure S7. Changes in soil C:N:P stoichiometry of China’s cropland across soil profiles in different soil types over the past 40 years. a-d,** changes in soil C:N at 0-20 **(a)**, 20-40 **(b)**, 40-60 **(c)**, and 60-100 cm **(d)** in different soil types. **e-h,** changes in soil C:P at 0-20 **(e)**, 20-40 **(f)**, 40-60 **(g)**, and 60-100 cm **(h)** in different soil types. **i-l,** changes in soil N:P at 0-20 **(i)**, 20-40 **(j)**, 40-60 **(k)**, and 60-100 cm **(l)** in different soil types. Box spans the 25th–75th percentiles, solid line indicates the median, filled dot in box denotes the mean. The significant differences between soil types are represented by different lowercase letters, *p* < 0.05.


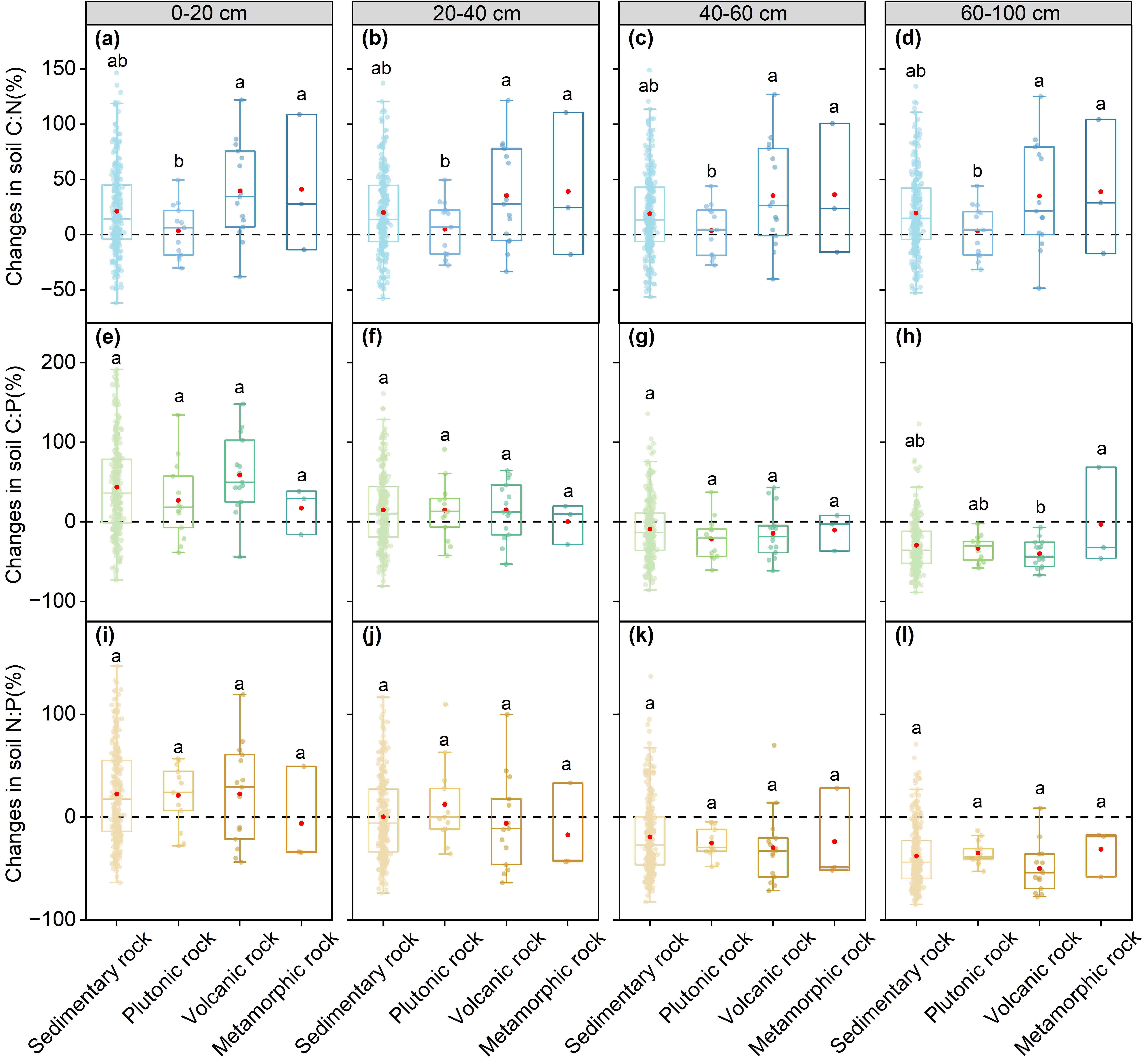


**Figure S8. Changes in soil C:N:P stoichiometry of China’s cropland across soil profiles in different parent material types over the past 40 years.** **a-d,** changes in soil C:N at 0-20 **(a)**, 20-40 **(b)**, 40-60 **(c)**, and 60-100 cm **(d)** in different parent material types. **e-h,** changes in soil C:P at 0-20 **(e)**, 20-40 **(f)**, 40-60 **(g)**, and 60-100 cm **(h)** in different parent material types. **i-l,** changes in soil N:P at 0-20 **(i)**, 20-40 **(j)**, 40-60 **(k)**, and 60-100 cm **(l)** in different parent material types. Box spans the 25th–75th percentiles, solid line indicates the median, filled dot in box denotes the mean. The significant differences between parent material types are represented by different lowercase letters, *p* < 0.05.


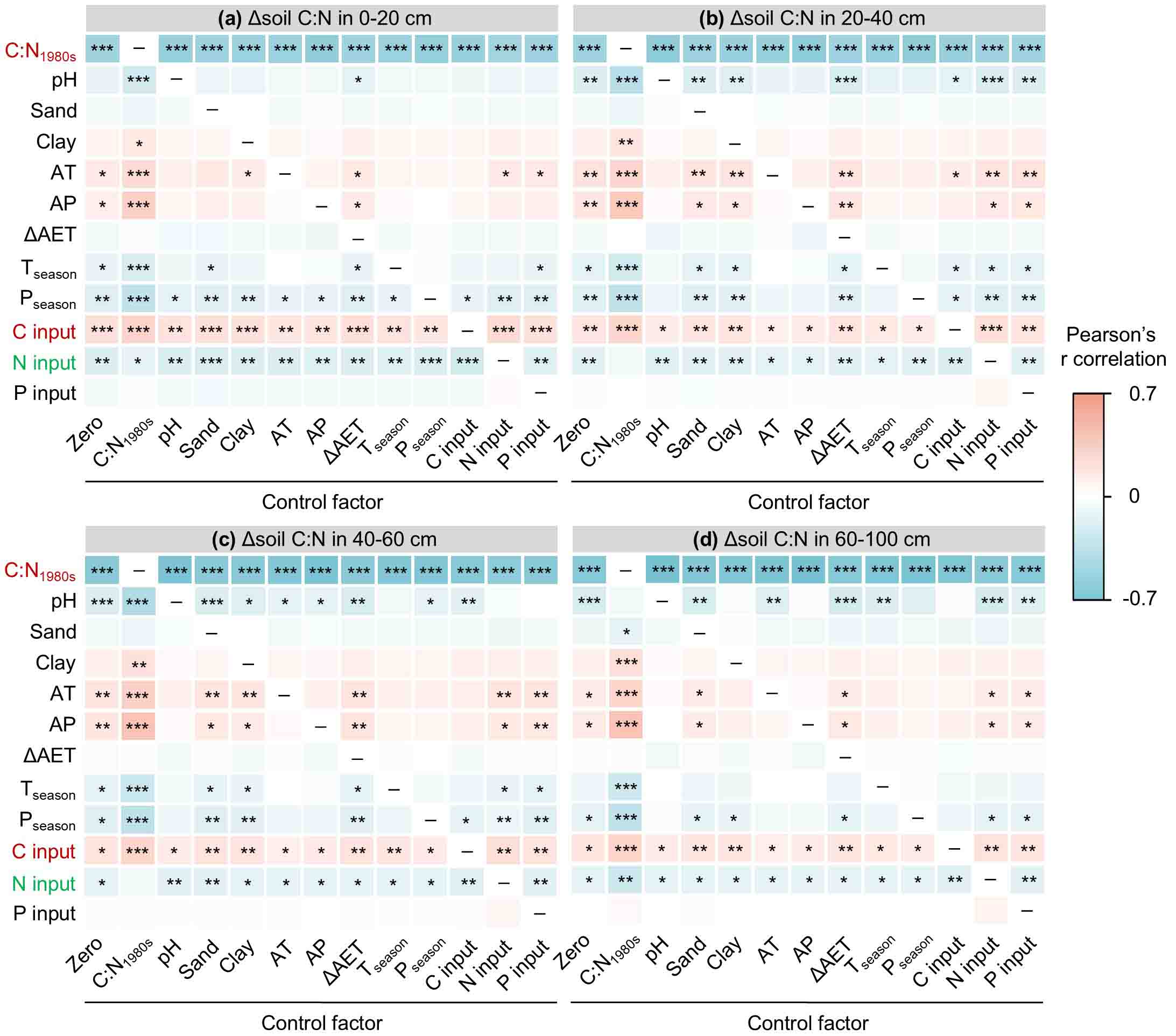


**Figure S9.** **Partial correlations between changes in soil C:N stoichiometry and predictors.** The x-axis represents the zero-order correlation (uncontrolled) and the controlled factor. The y-axis represents factors. Colors indicate correlation strength. Differences in color between zero-order and controlled factors reflect the dependence of soil C:N changes correlations on the controlled variable (no color change = no dependence; decreased/increased intensity = weakened/strengthened correlation). **p* < 0.05,***p* < 0.01,****p* < 0.001. C:N_1980s_, background (1980s) soil C:N stoichiometry; AP, accumulated precipitation; AT, accumulated temperature; ΔAET, changes in actual evapotranspiration from 1981–1985 (average) to 2019–2023 (average); T_season_, temperature seasonality; P_season_, precipitation seasonality; C input, N input, and P input, the cumulative C, N, and P inputs over the last 40 years, respectively.

**
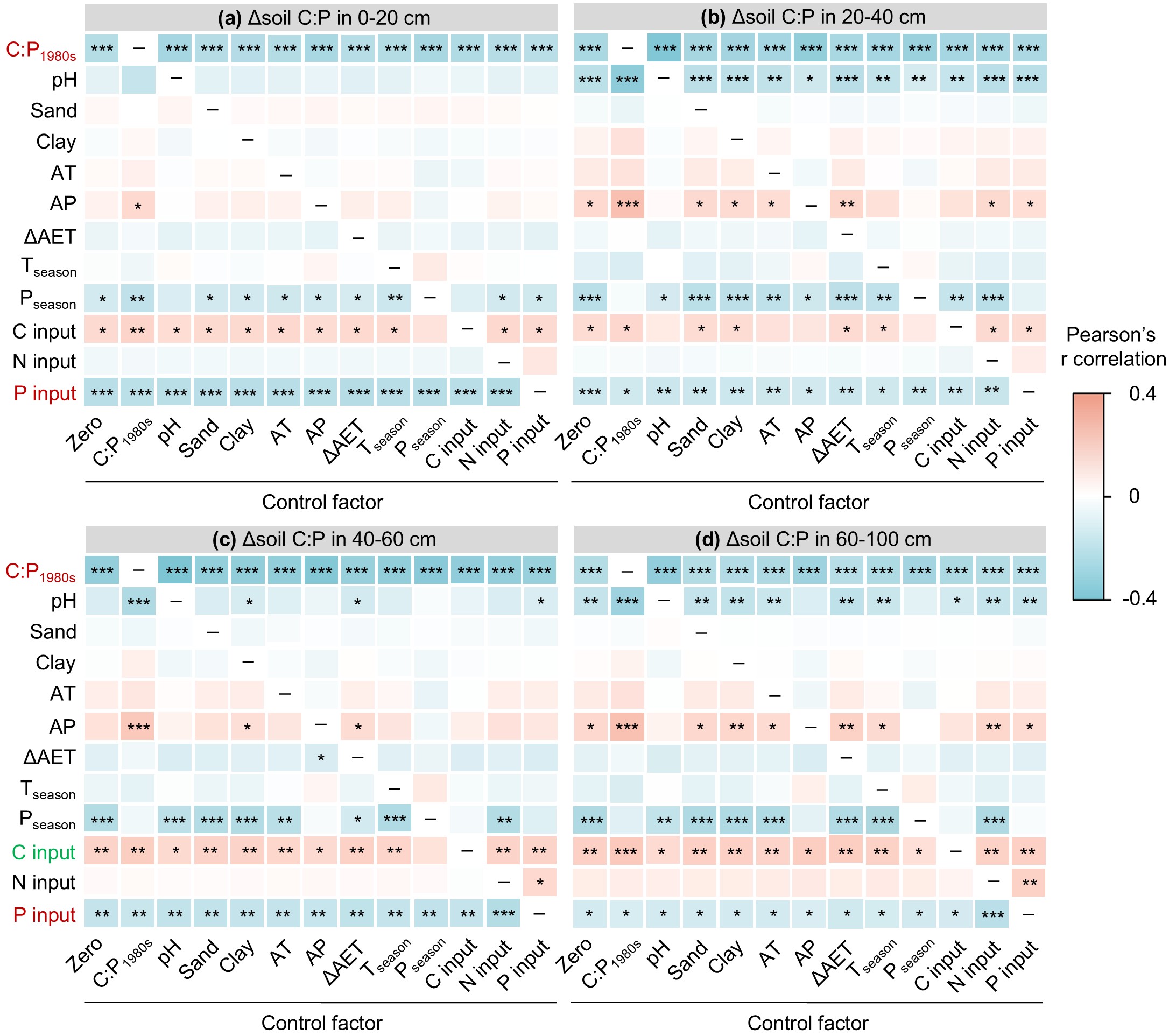
**

**Figure S10. Partial correlations between changes in soil C:P stoichiometry and predictors.** The x-axis represents the zero-order correlation (uncontrolled) and the controlled factor. The y-axis represents factors. Colors indicate correlation strength. Differences in color between zero-order and controlled factors reflect the dependence of soil C:P changes correlations on the controlled variable (no color change = no dependence; decreased/increased intensity = weakened/strengthened correlation). **p* < 0.05,***p* < 0.01,****p* < 0.001. C:P_1980s_, background (1980s) soil C:P stoichiometry; AP, accumulated precipitation; AT, accumulated temperature; ΔAET, changes in actual evapotranspiration from 1981–1985 (average) to 2019–2023 (average); T_season_, temperature seasonality; P_season_, precipitation seasonality; C input, N input, and P input, the cumulative C, N, and P inputs over the last 40 years, respectively.

**
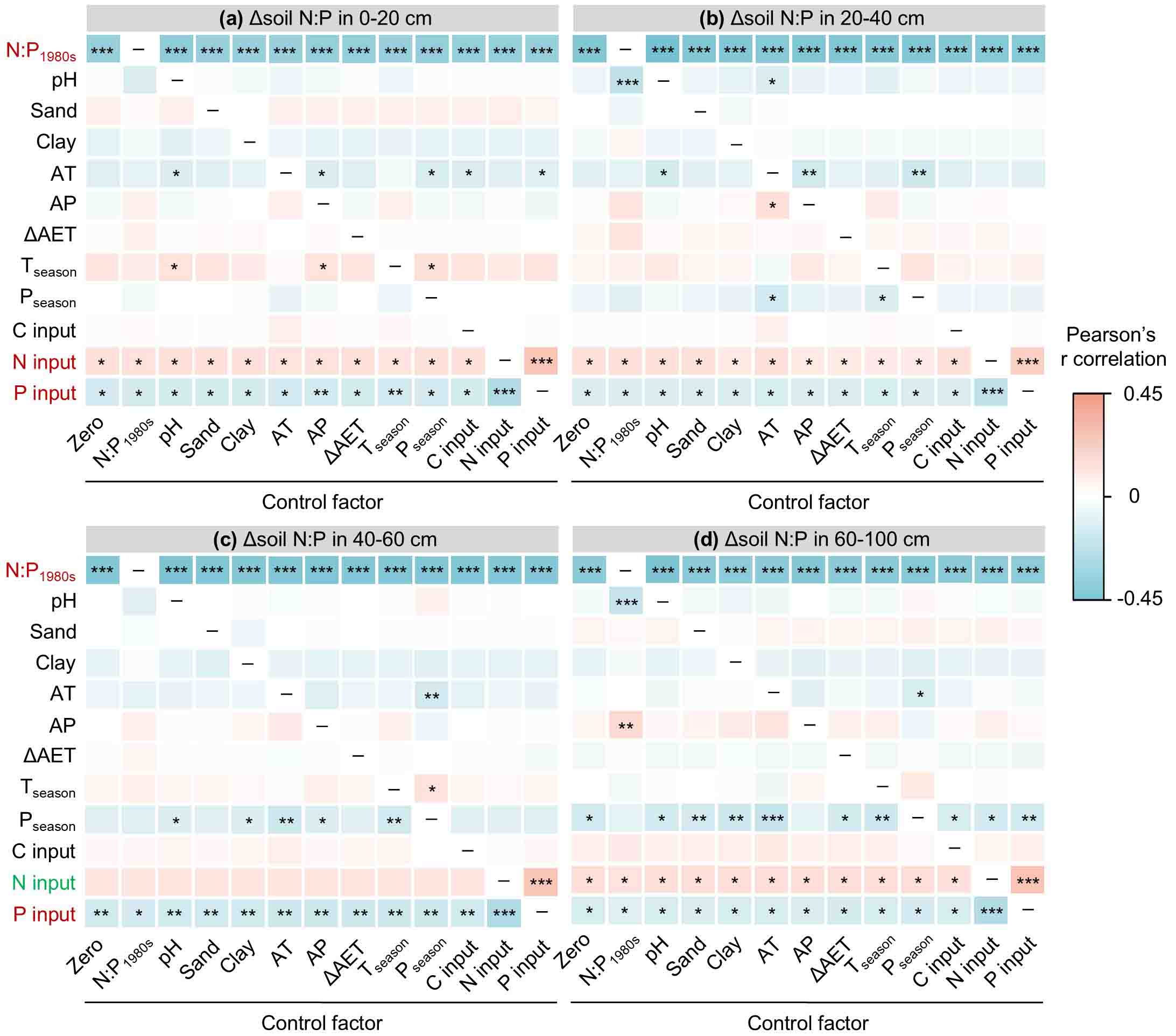
**

**Figure S11. Partial correlations between changes in soil N:P stoichiometry and predictors.** The x-axis represents the zero-order correlation (uncontrolled) and the controlled factor. The y-axis represents factors. Colors indicate correlation strength. Differences in color between zero-order and controlled factors reflect the dependence of soil N:P changes correlations on the controlled variable (no color change = no dependence; decreased/increased intensity = weakened/strengthened correlation). **p* < 0.05,***p* < 0.01,****p* < 0.001. N:P_1980s_, background (1980s) soil N:P stoichiometry; AP, accumulated precipitation; AT, accumulated temperature; ΔAET, changes in actual evapotranspiration from 1981–1985 (average) to 2019–2023 (average); T_season_, temperature seasonality; P_season_, precipitation seasonality; C input, N input, and P input, the cumulative C, N, and P inputs over the last 40 years, respectively.

**
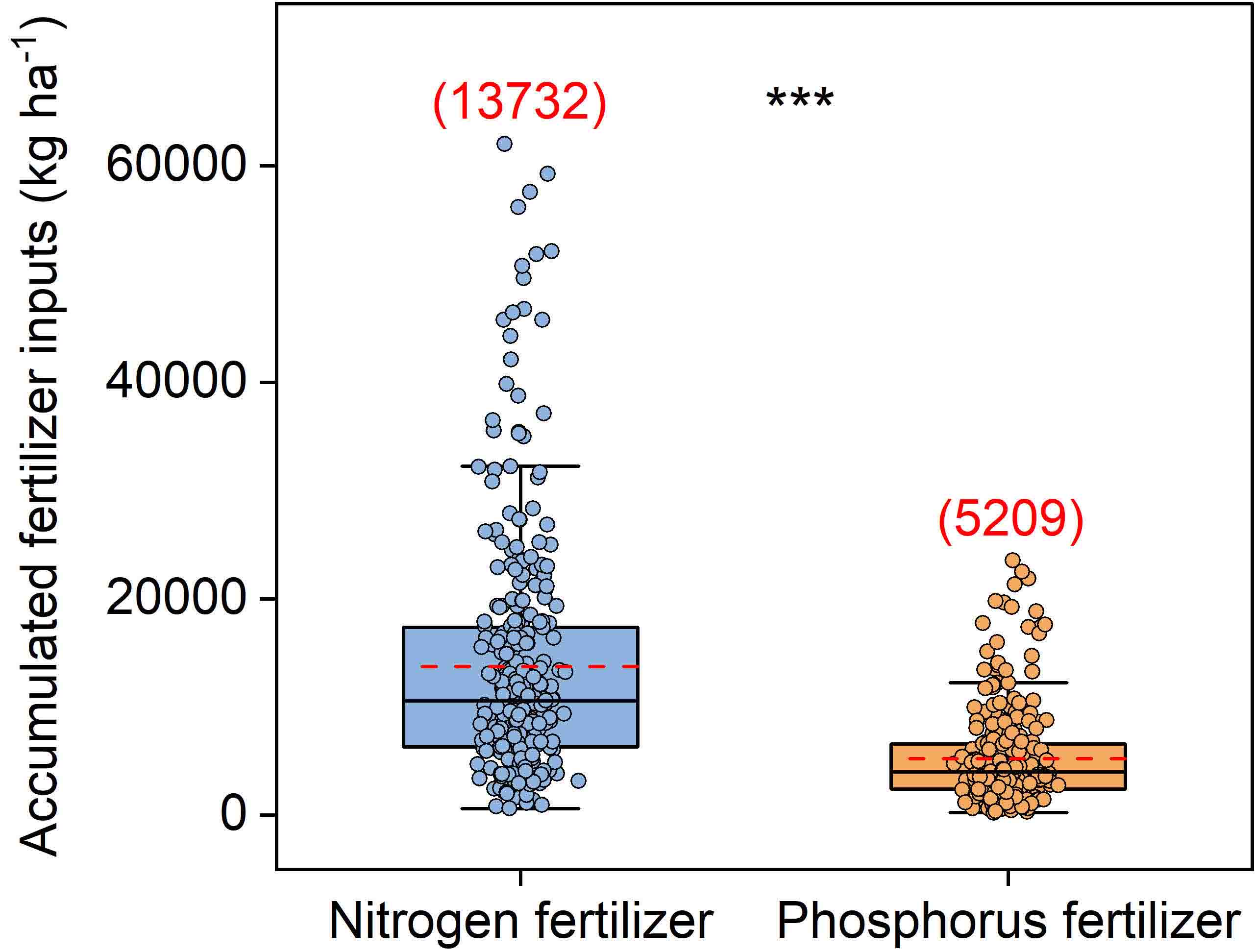
**

**Figure S12. Accumulated nitrogen and phosphorus fertilizer inputs over the past four decades.** Central line and whiskers (red) in each box represent the median and mean (n=305), respectively. Boxes indicate the interquartile range between 25th and 75th percentile. ****p*< 0.001 according to paired-sample t tests.


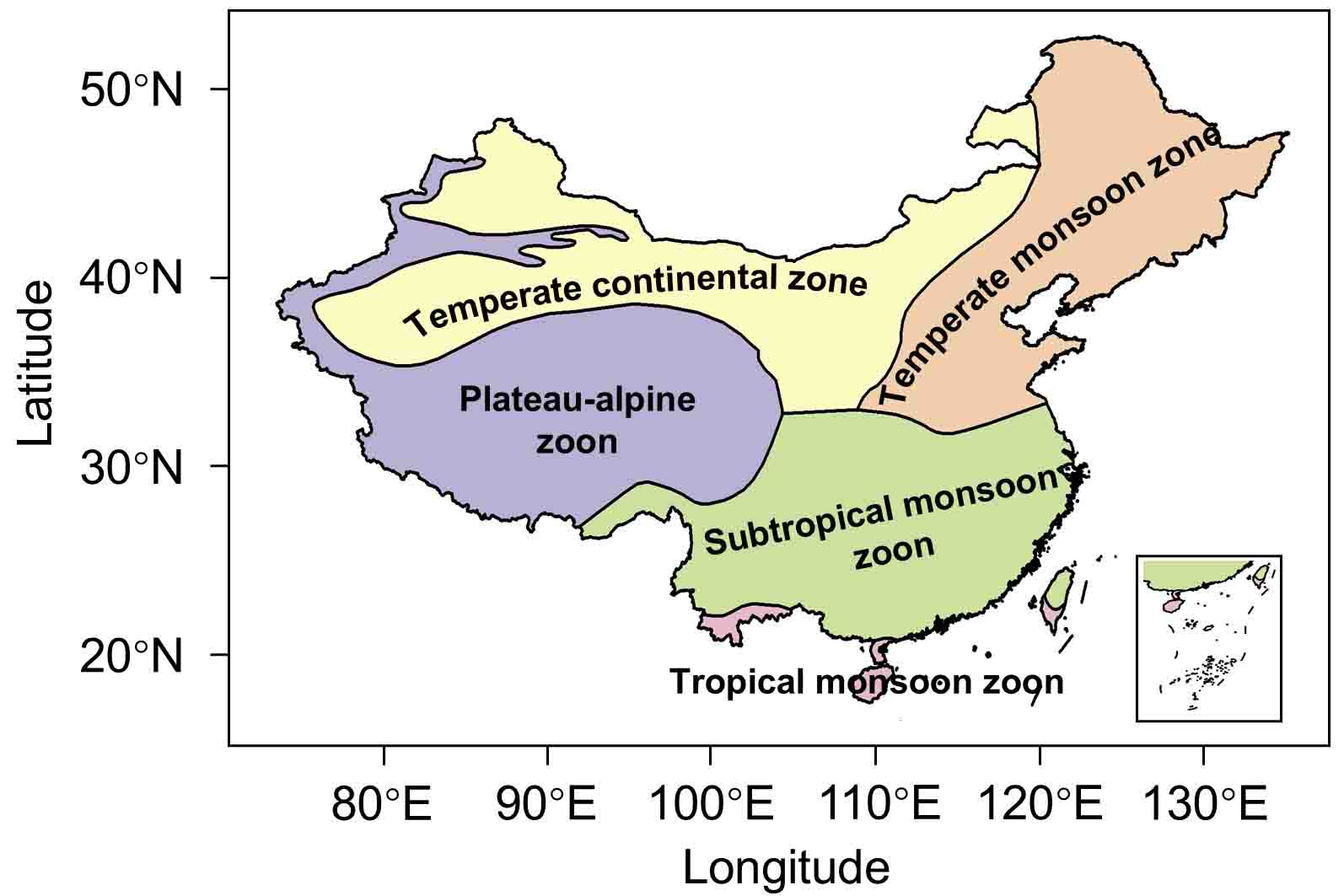


**Figure S13. Distribution of five major climate zones in China.**

**Table S1. Performance metrics of random-forest models for changes in soil C:N:P stoichiometry across depths.**

| Response | Depth (cm) | n | OOB *R*^2^  (% Var explained) | NRMSE | CV(RMSE) |
| --- | --- | --- | --- | --- | --- |
| ΔC:N | 0–20 | 305 | 40.89% | 0.14 | 0.77 |
| ΔC:N | 20–40 | 305 | 41.25% | 0.15 | 0.77 |
| ΔC:N | 40–60 | 305 | 48.04% | 0.13 | 0.72 |
| ΔC:N | 60–100 | 305 | 46.86% | 0.14 | 0.73 |
| ΔC:P | 0–20 | 305 | 15.07% | 0.20 | 0.92 |
| ΔC:P | 20–40 | 305 | 15.14% | 0.17 | 0.92 |
| ΔC:P | 40–60 | 305 | 19.23% | 0.15 | 0.90 |
| ΔC:P | 60–100 | 305 | 13.94% | 0.13 | 0.93 |
| ΔN:P | 0–20 | 305 | 17.13% | 0.19 | 0.91 |
| ΔN:P | 20–40 | 305 | 22.54% | 0.19 | 0.88 |
| ΔN:P | 40–60 | 305 | 23.36% | 0.14 | 0.87 |
| ΔN:P | 60–100 | 305 | 23.77% | 0.16 | 0.87 |

n, number of observations; *R*^2^, coefficient of determination based on out-of-bag (OOB) predictions, equivalent to % Var explained in the randomForest output; RMSE, root mean square error; NRMSE, RMSE normalized by the observed range; CV(RMSE), RMSE expressed relative to the standard deviation.

**Table S2. Uncertainty and sensitivity analysis across soil depth layers.**

| Depth (cm) | Metric | Mean [95%CI] (%) | Directional stability (%) | Boundary Δmean | Boundary Δmean/halfCI | Boundary impact flag |
| --- | --- | --- | --- | --- | --- | --- |
| 0-20 | ΔSOC | 59.57 [46.91, 71.99] | 93.611 | 0.141 | 0.019 | ≈0 |
|  | ΔTN | 40.89 [36.96, 76.30] | 80.525 | 0.029 | 0.019 | ≈0 |
|  | ΔTP | 27.41 [20.33, 30.25] | 88.035 | 0.006 | 0.006 | ≈0 |
|  | ΔC:N | 24.16 [16.67, 31.83] | 81.947 | -0.172 | -0.017 | ≈0 |
|  | ΔC:P | 46.07 [35.51, 54.26] | 80.129 | 0.348 | 0.018 | ≈0 |
|  | ΔN:P | 24.43 [17.82, 29.79] | 83.295 | 0.195 | 0.015 | ≈0 |
| 20-40 | ΔSOC | 43.21 [34.27, 52.56] | 87.477 | -0.024 | -0.002 | ≈0 |
|  | ΔTN | 22.59 [13.25, 33.57] | 87.135 | -0.003 | -0.003 | ≈0 |
|  | ΔTP | 38.42 [28.74, 49.20] | 91.897 | -0.001 | -0.001 | ≈0 |
|  | ΔC:N | 21.34 [15.07, 28.48] | 86.35 | 0.008 | 0.001 | ≈0 |
|  | ΔC:P | 24.80 [20.93, 29.64] | 92.204 | -0.05 | 0 | ≈0 |
|  | ΔN:P | 4.44 [1.82, 9.18] | 90.345 | -0.008 | -0.001 | ≈0 |
| 40-60 | ΔSOC | 31.50 [21.41, 41.48] | 88.367 | -0.007 | -0.001 | ≈0 |
|  | ΔTN | 16.89 [10.51, 24.72] | 84.087 | -0.001 | -0.002 | ≈0 |
|  | ΔTP | 56.13 [43.46, 67.39] | 86.652 | 0 | 0 | ≈0 |
|  | ΔC:N | 20.97 [18.71, 23.54] | 85.801 | 0.026 | 0.002 | ≈0 |
|  | ΔC:P | -16.97 [-29.11, -5.57] | 91.791 | 0.06 | 0 | ≈0 |
|  | ΔN:P | -25.56 [-33.18, -19.39] | 91.365 | 0.013 | 0.001 | ≈0 |
| 60-100 | ΔSOC | 25.00 [22.32, 28.73] | 88.424 | -0.006 | 0 | ≈0 |
|  | ΔTN | 10.34 [8.34, 16.84] | 80.838 | -0.001 | -0.001 | ≈0 |
|  | ΔTP | 87.78 [80.91, 92.96] | 84.469 | 0 | 0 | ≈0 |
|  | ΔC:N | 21.26 [14.24, 29.60] | 86.992 | -0.005 | 0 | ≈0 |
|  | ΔC:P | -27.37 [-33.59, -23.09] | 90.875 | 0.044 | 0.006 | ≈0 |
|  | ΔN:P | -43.99 [-49.38, -30.02] | 87.334 | 0.152 | 0.008 | ≈0 |

Mean [95%CI] (%), average relative change with bootstrap 95% confidence interval; Directional stability (%), proportion of bootstrap replicates with the same sign as the mean (robustness of direction); Boundary Δmean, mean shift under ±2 cm perturbation of horizon boundaries; Boundary Δmean/halfCI, boundary effect scaled to half the bootstrap CI width (sensitivity index); Boundary impact flag, qualitative indicator (≈0, negligible impact).

**Table S3. The fitted equation of SOC, TN, and TP change with soil depth for each soil profile in the 1980s.**

|  | **SOC** | | **TN** | | **TP** | |
| --- | --- | --- | --- | --- | --- | --- |
| **ID** | Model expression | *R*^2^ | Model expression | *R*^2^ | Model expression | *R*^2^ |
| 1 | *y*=54.86*x*^-0.59^ | 0.973 | *y*=7.55*x*^-0.6390^ | 0.991 | *y*=0.95-0.1421ln*x* | 0.957 |
| 2 | *y*=13.90e^-0.01^*^x^* | 0.995 | *y* = 1.20*x*^-0.0645^ | 0.906 | *y*=0.48-0.0029*x* | 0.998 |
| 3 | *y*=7.77-0.10*x* | 1.000 | *y*=4.37*x*^-0.7289^ | 0.993 | *y*=0.32-0.0030*x* | 0.996 |
| 4 | *y*=10.07-1.92ln*x* | 1.000 | *y*=0.49-0.0028*x* | 0.936 | *y*=0.34e^-0.0028^*^x^* | 1.000 |
| 5 | *y*=21.33*x*^-0.32^ | 1.000 | *y*=1.47-0.1641ln*x* | 0.998 | *y*=0.83-0.0015x | 0.998 |
| 6 | *y*=8.60e^-0.01^*^x^* | 1.000 | *y*=1.25-0.0130*x* | 0.998 | *y*=0.16e^0.0184^*^x^* | 0.989 |
| 7 | *y*=20.26-0.20*x* | 0.995 | *y*=3.00-0.5171ln*x* | 1.000 | *y*=2.37*x*^-0.5397^ | 1.000 |
| 8 | *y*=13.09-0.13*x* | 0.947 | *y*=1.21-0.0109*x* | 0.964 | *y*=0.48-0.0037*x* | 0.934 |
| 9 | *y*=60.58-0.32*x* | 0.861 | *y*=4.07-0.0249*x* | 0.980 | *y*=0.50-0.0014*x* | 0.975 |
| 10 | *y*=14.39-2.60ln*x* | 0.999 | *y*=1.30*x*^-0.2202^ | 0.858 | *y*=0.59-0.0660ln*x* | 1.000 |
| 11 | *y*=19.42*x*^-0.48^ | 0.997 | *y*=5.99*x*^-0.5940^ | 0.954 | *y*=2.59*x*^-0.7902^ | 0.999 |
| 12 | *y*=16.04e^-0.01^*^x^* | 1.000 | *y*=1.25-0.0073*x* | 0.993 | *y*=0.69+0.0026*x* | 0.999 |
| 13 | *y*=12.59-2.37ln*x* | 0.998 | *y*=1.33-0.2267ln*x* | 1.000 | *y*=1.09*x*^-0.3342^ | 0.990 |
| 14 | *y*=93.09*x*^-1.06^ | 0.993 | *y*=26.08*x*^-1.5498^ | 0.970 | *y*=0.96*x*^-0.2301^ | 0.944 |
| 15 | *y*=10.89*x*^-0.19^ | 0.961 | *y*=2.53*x*^-0.4360^ | 0.907 | *y*=1.01-0.0043*x* | 0.970 |
| 16 | *y*=10.49-0.10*x* | 0.998 | *y*=1.55e^-0.0234^*^x^* | 0.999 | *y*=0.74-0.0060*x* | 0.952 |
| 17 | *y*=15.77-2.74ln*x* | 1.000 | *y*=1.17-0.0099*x* | 0.993 | *y*=0.42-0.0027*x* | 0.986 |
| 18 | *y*=12.78-0.11*x* | 0.934 | *y*=1.30-0.0084*x* | 0.979 | *y*=1.10-0.1424ln*x* | 0.999 |
| 19 | *y*=11.76-0.10*x* | 0.946 | *y*=1.47-0.0126*x* | 0.963 | *y*=0.92-0.0063*x* | 0.940 |
| 20 | *y*=23.23e^-0.02^*^x^* | 1.000 | *y*=1.44-0.0112*x* | 1.000 | *y*=0.24+0.0237ln*x* | 0.867 |
| 21 | *y*=149.97*x*^-1.06^ | 0.991 | *y*=6.08*x*^-0.7753^ | 0.999 | *y*=2.03*x*^-0.6795^ | 0.982 |
| 22 | *y*=32.22e^-0.02^*^x^* | 1.000 | *y*=1.32-0.0102*x* | 0.995 | *y*=2.42*x*^-0.4845^ | 0.947 |
| 23 | *y*=3.51-0.02*x* | 0.882 | *y*=0.50-0.0029*x* | 0.907 | *y*=0.58-0.0016*x* | 0.860 |
| 24 | *y*=6.57e^-0.03^*^x^* | 0.956 | *y*=1.32-0.2602ln*x* | 0.928 | *y*=0.66-0.0027*x* | 0.852 |
| 25 | *y*=12.35*x*^-0.42^ | 0.914 | *y*=0.78-0.0066*x* | 0.981 | *y*=1.01-0.1563ln*x* | 0.949 |
| 26 | *y*=14.80-2.68ln*x* | 0.946 | *y*=1.97-0.3736ln*x* | 0.910 | *y*=0.58-0.0012*x* | 0.908 |
| 27 | *y*=3.93-0.03*x* | 0.994 | *y*=0.53e^-0.0154^*^x^* | 0.994 | *y*=0.58-0.0032*x* | 0.996 |
| 28 | *y*=6.52-0.04*x* | 0.935 | *y*=0.77-0.0039*x* | 0.861 | *y*=0.45*x*^0.0968^ | 0.995 |
| 29 | *y*=8.41e^-0.02^*^x^* | 1.000 | *y*=1.92-0.3885ln*x* | 0.999 | *y*=0.86*x*^-0.0723^ | 0.979 |
| 30 | *y*=3.79-0.03*x* | 0.959 | *y*=0.69-0.0034*x* | 0.964 | *y*=0.63-0.0036*x* | 0.878 |
| 31 | *y*=70.37*x*^-0.74^ | 0.996 | *y*=2.04-0.3988ln*x* | 0.994 | *y*=0.86-0.0020*x* | 0.884 |
| 32 | *y*=10.26*x*^-0.20^ | 0.963 | *y*=4.85*x*^-0.4997^ | 0.991 | *y*=0.59-0.0015*x* | 0.964 |
| 33 | *y*=5.99-0.03*x* | 0.968 | *y*=0.81-0.0041*x* | 0.874 | *y*=-0.08+0.1553ln*x* | 0.924 |
| 34 | *y*=6.53-0.10*x* | 0.874 | *y*=0.50-0.0069*x* | 0.997 | *y*=0.49-0.0058*x* | 0.987 |
| 35 | *y*=11.96-0.11*x* | 0.988 | *y*=1.48e^-0.0171^*^x^* | 1.000 | *y*=0.31-7e-04*x* | 0.988 |
| 36 | *y*=6.00-1.03ln*x* | 0.997 | *y*=0.94*x*^-0.3260^ | 1.000 | *y*=0.20e^-0.0089^*^x^* | 1.000 |
| 37 | *y*=8.24e^-0.01^*^x^* | 0.999 | *y*=0.95e^-0.0155^*^x^* | 1.000 | *y*=1.06*x*^-0.1978^ | 0.906 |
| 38 | *y*=43.41*x*^-1.00^ | 0.929 | *y*=1.23-0.2439ln*x* | 0.987 | *y*=0.36e^-0.0143^*^x^* | 0.994 |
| 39 | *y*=3.77-0.02*x* | 0.911 | *y*=0.45-0.0029*x* | 0.977 | *y*=0.39e^0.0087^*^x^* | 0.944 |
| 40 | *y*=13.12-2.68ln*x* | 0.998 | *y*=1.43-0.2928ln*x* | 1.000 | *y*=1.03*x*^-0.1397^ | 0.955 |
| 41 | *y*=6.06-0.06*x* | 0.992 | *y*=0.67-0.0061*x* | 0.989 | *y*=0.43-0.0035*x* | 0.984 |
| 42 | *y*=41.92*x*^-0.72^ | 0.930 | *y*=1.52-0.2880ln*x* | 0.984 | *y*=0.82-0.1722ln*x* | 0.999 |
| 43 | *y*=7.96-0.05*x* | 0.971 | *y*=1.95-0.3576ln*x* | 0.988 | *y*=0.62-0.0032*x* | 0.915 |
| 44 | *y*=800.78*x*-1.24 | 0.986 | *y*=17.47x^-0.8605^ | 0.969 | *y*=2.88*x*^-0.6662^ | 0.856 |
| 45 | *y*=25.98*x*-0.43 | 0.941 | *y*=1.58-0.2409ln*x* | 0.962 | *y*=0.45e^0.0039^*^x^* | 0.871 |
| 46 | *y*=11.14-1.67ln*x* | 0.886 | *y*=1.26-0.2026ln*x* | 0.854 | *y*=0.58-0.0033*x* | 0.984 |
| 47 | *y*=4.89-0.02*x* | 1.000 | *y*=0.58-0.0013*x* | 0.929 | *y*=0.50-0.0015*x* | 0.907 |
| 48 | *y*=1.63-0.01*x* | 0.997 | *y*=0.64-0.1169ln*x* | 0.993 | *y*=1.46-0.2800ln*x* | 0.997 |
| 49 | *y*=9.89-1.28ln*x* | 0.986 | *y*=1.54-0.2426ln*x* | 0.895 | *y*=0.71*x*^-0.1875^ | 0.872 |
| 50 | *y*=15.95-2.67ln*x* | 0.998 | *y*=1.56-0.2489ln*x* | 0.988 | *y*=0.60-0.0038*x* | 0.967 |
| 51 | *y*=141.93*x*^-0.97^ | 0.860 | *y*=2.25-0.4567ln*x* | 0.898 | *y*=0.07*x*^-0.4603^ | 0.980 |
| 52 | *y*=21.62*x*^-0.47^ | 1.000 | *y* =0.68-0.0033*x* | 0.962 | *y*=0.82-0.0026*x* | 0.957 |
| 53 | *y*=9.99-1.29ln*x* | 0.958 | *y*=1.17-0.1464ln*x* | 0.874 | *y*=1.21-0.2132ln*x* | 0.968 |
| 54 | *y*=6.14e^-0.01^*^x^* | 0.960 | *y*=0.81-0.0034*x* | 0.967 | *y*=0.23-0.0178ln*x* | 0.880 |
| 55 | *y*=14.91*x*^-0.14^ | 0.955 | *y*=1.86*x*^-0.1792^ | 1.000 | *y*=0.63-4e-04*x* | 0.910 |
| 56 | *y*=14.35e^-0.01^*^x^* | 0.989 | *y*=9.12*x*^-0.6117^ | 0.919 | *y*=-0.63+0.2623ln*x* | 0.956 |
| 57 | *y*=8.95-0.07*x* | 0.988 | *y*=0.79-0.0056*x* | 0.996 | y=1.54-0.2416ln*x* | 0.915 |
| 58 | *y*=16.61-3.10ln*x* | 0.869 | *y*=4.54*x*^-0.5324^ | 0.901 | *y*=0.43e^0.0032^*^x^* | 0.988 |
| 59 | *y*=9.86-0.08*x* | 0.996 | *y*=1.05-0.0082*x* | 0.993 | *y*=4.97*x*^-0.7008^ | 0.997 |
| 60 | *y*=14.93-2.64ln*x* | 0.984 | *y*=1.21-0.1713ln*x* | 0.933 | *y*=0.81e^-0.0049^*^x^* | 0.995 |
| 61 | *y*=46.08*x*^-0.60^ | 1.000 | *y*=3.81*x*^-0.5520^ | 1.000 | *y*=2.64*x*^-0.3652^ | 0.894 |
| 62 | *y*=476.90*x*^-1.11^ | 0.932 | *y*=56.98*x*^-1.1645^ | 0.942 | *y*=0.84e^-0.0098^*^x^* | 0.981 |
| 63 | *y*=232.46*x*^-0.98^ | 0.970 | *y*=8.32*x*^-0.6587^ | 0.963 | *y*=0.56e^0.0040^*^x^* | 0.947 |
| 64 | *y*=6.21-0.04*x* | 0.855 | *y*=0.75-0.0051*x* | 0.977 | *y*=0.53e^8e-04^*^x^* | 0.996 |
| 65 | *y* =172.83*x*^-1.01^ | 0.931 | *y*=2.14-0.4135ln*x* | 0.876 | *y*=1.90*x*^-0.6118^ | 0.880 |
| 66 | *y*=2066.57*x*^-1.79^ | 0.956 | *y*=1.59*x*^-0.5439^ | 1.000 | *y*=0.43-0.0024*x* | 0.993 |
| 67 | *y*=30.84-6.37ln*x* | 0.998 | *y*=2.88-0.5774ln*x* | 0.991 | *y*=1.16-0.1180ln*x* | 0.915 |
| 68 | *y*=11.21-0.07*x* | 0.964 | *y*=1.23-0.0102*x* | 0.997 | *y*=0.79-0.0020*x* | 0.984 |
| 69 | *y*=34.15*x*^-0.51^ | 0.964 | *y*=3.07*x*^-0.4164^ | 0.992 | *y*=1.23-0.2188ln*x* | 0.887 |
| 70 | *y*=7.21-0.06*x* | 0.996 | *y*=0.72-0.0063*x* | 0.976 | *y*=0.52e^-0.0112^*^x^* | 0.982 |
| 71 | *y*=7.75-0.05*x* | 0.935 | *y*=0.79-0.0050*x* | 0.984 | *y*=0.55e-0.0013*x* | 0.891 |
| 72 | *y*=6.58e^-0.01^*^x^* | 0.999 | *y*=0.82e-0.0088*x* | 0.999 | *y*=0.07+0.0730ln*x* | 0.999 |
| 73 | *y*=143.72*x*^-0.96^ | 0.899 | *y*=1.64-0.2850ln*x* | 0.848 | *y*=0.68e^-0.0202^*^x^* | 0.962 |
| 74 | *y*=12.90*x*^-0.34^ | 0.929 | *y*=2.83*x*^-0.5001^ | 0.890 | *y*=0.48+0.0037*x* | 0.991 |
| 75 | *y*=18.54-3.10ln*x* | 0.869 | *y*=1.70-0.2479ln*x* | 0.857 | *y*=1.30-0.2149ln*x* | 0.869 |
| 76 | *y*=6.58-0.05*x* | 0.913 | *y*=0.66-0.0045*x* | 1.000 | *y*=0.48e^0.0064^*^x^* | 0.996 |
| 77 | *y*=14.61-2.92ln*x* | 0.997 | *y*=0.85e^-0.0172^*^x^* | 0.995 | *y*=0.74*x*^-0.0680^ | 0.957 |
| 78 | *y*=6.85e^-0.02^*^x^* | 1.000 | *y*=0.74e^-0.0165^*^x^* | 0.999 | *y*=0.67-0.0014*x* | 1.000 |
| 79 | *y*=19.07-4.06ln*x* | 1.000 | *y*=9.58*x*^-0.9045^ | 0.852 | *y*=0.32e^-0.0035^*^x^* | 1.000 |
| 80 | *y*=27.79*x*^-0.52^ | 0.852 | *y*=2.69*x*^-0.4520^ | 0.966 | *y*=0.85+-0.0056*x* | 0.972 |
| 81 | *y*=6.19-0.04*x* | 0.996 | *y*=0.77-0.0045*x* | 0.914 | *y*=0.06e^-0.0160^*^x^* | 0.967 |
| 82 | *y*=80.17*x*^-0.57^ | 0.904 | *y*=6.64*x*-0.4925 | 0.893 | *y*=2.87*x*-0.5118 | 0.983 |
| 83 | *y*=10.04e^-0.01^*^x^* | 0.966 | *y*=1.04e-0.0098*x* | 0.975 | *y*=1.30-0.2498ln*x* | 0.991 |
| 84 | *y*=6.89-0.06*x* | 0.989 | *y*=1.04-0.0085*x* | 0.999 | *y*=0.48-0.0038*x* | 0.999 |
| 85 | *y*=10.14e^-0.01^*^x^* | 0.995 | *y*=1.64x^-0.2987^ | 0.948 | *y*=2.81-0.3221ln*x* | 0.953 |
| 86 | *y*=7.12-1.39ln*x* | 0.999 | *y*=0.51-0.0010*x* | 0.866 | *y*=1.38-0.0125*x* | 0.991 |
| 87 | *y*=71.85*x*^-0.59^ | 0.919 | *y*=5.41*x*^-0.5444^ | 0.927 | *y*=0.93-0.0055*x* | 0.998 |
| 88 | *y* =3.52-0.01*x* | 1.000 | *y*=1.38*x*^-0.3658^ | 1.000 | *y*=0.53e^-0.0019^*^x^* | 1.000 |
| 89 | *y*=6.71-0.03*x* | 1.000 | *y*=0.90-0.0043*x* | 0.999 | *y*=0.58-0.0029*x* | 0.999 |
| 90 | *y*=8.72-1.37ln*x* | 0.885 | *y*=0.92-0.1215ln*x* | 0.919 | *y*=0.64-0.0607ln*x* | 0.984 |
| 91 | *y*=5.33-0.02*x* | 0.875 | *y*=0.58-0.0023*x* | 0.917 | *y*=0.45e^-0.0045^*^x^* | 1.000 |
| 92 | *y*=55.62-10.37ln*x* | 0.978 | *y*=23.58*x*^-0.8509^ | 0.995 | *y*=0.29e^0.0120^*^x^* | 0.956 |
| 93 | *y*=9.62e^-0.02^*^x^* | 0.995 | *y*=0.81-0.0068*x* | 0.992 | *y*=0.73-0.0044*x* | 0.940 |
| 94 | *y*=20.04-0.11*x* | 0.958 | *y*=5.05*x*^-0.3741^ | 0.872 | *y*=0.55+0.0848ln*x* | 0.941 |
| 95 | *y*=47.22*x*^-0.51^ | 0.852 | *y*=1.13-0.0060*x* | 0.994 | *y*=0.37e^0.0060^*^x^* | 0.955 |
| 96 | *y*=39.14*x*^-0.68^ | 0.953 | *y*=1.29-0.1889ln*x* | 0.903 | *y*=0.50-0.0025*x* | 0.989 |
| 97 | *y*=47.21*x*^-0.62^ | 0.950 | *y*=3.33*x*^-0.4912^ | 0.931 | *y*=0.19e^0.0089^*^x^* | 0.854 |
| 98 | *y*=12.99-0.06*x* | 0.911 | *y*=1.19-0.0038*x* | 0.894 | *y*=0.10e^0.0084^*^x^* | 0.941 |
| 99 | *y*=11.37-0.07*x* | 0.980 | *y*=1.61-0.0123*x* | 0.980 | *y*=0.66-0.0045*x* | 0.941 |
| 100 | *y*=10.22e^-0.01^*^x^* | 0.941 | *y*=0.91-0.0042*x* | 0.960 | *y*=0.80-0.1503ln*x* | 0.997 |
| 101 | *y*=11.59-2.16ln*x* | 1.000 | *y*=0.91-0.0065*x* | 0.999 | *y*=0.28-0.0010*x* | 0.859 |
| 102 | *y*=9.86e^-0.01^*^x^* | 0.973 | *y*=0.95e^-0.0078^*^x^* | 0.983 | *y*=0.26-0.0010*x* | 0.996 |
| 103 | *y*=23.45-4.24ln*x* | 0.985 | *y*=15.30*x*^-0.8718^ | 0.988 | *y*=0.99-0.1314ln*x* | 0.947 |
| 104 | *y*=30.50-0.14*x* | 0.862 | *y*=22.08*x*^-0.7426^ | 1.000 | *y*=0.88 e^0.0082^*^x^* | 0.917 |
| 105 | *y*=13.05-0.08*x* | 0.921 | *y*=1.21-0.0083*x* | 0.983 | *y*=0.28e^0.0091^*^x^* | 0.993 |
| 106 | *y*=652.74*x*^-1.12^ | 0.867 | *y*=23.10*x*^-0.8020^ | 0.962 | *y*=3.59*x*^-0.5796^ | 0.869 |
| 107 | *y*=18.44e^-0.01^*^x^* | 0.875 | *y*=3.14-0.5402ln*x* | 0.874 | *y*=0.66e^-0.0111^*^x^* | 0.924 |
| 108 | *y*=18.31-3.52ln*x* | 0.992 | *y*=7.80*x*^-0.7285^ | 0.988 | *y*=0.13-0.0010*x* | 0.992 |
| 109 | *y*=7.48-0.05*x* | 0.981 | *y*=3.24-0.6307ln*x* | 0.981 | *y*=0.23e^0.0090^*^x^* | 0.989 |
| 110 | *y*=1336.96*x*^-1.45^ | 0.956 | *y*=3.20-0.6059ln*x* | 0.948 | *y*=0.89*x*^-0.5754^ | 0.962 |
| 111 | *y*=7.25-0.05*x* | 0.988 | *y*=18.89*x*^-0.8804^ | 0.998 | *y*=0.36-0.0582ln*x* | 0.995 |
| 112 | *y*=599.99*x*^-1.33^ | 0.950 | *y*=105.56*x*^-1.3519^ | 0.975 | *y*=0.12e^0.0148^*^x^* | 0.980 |
| 113 | *y*=212.06*x*^-0.86^ | 1.000 | *y*=25.01*x*^-0.9077^ | 0.961 | *y*=0.32-0.0015*x* | 0.992 |
| 114 | *y*=16.67-3.24ln*x* | 0.861 | *y*=1.57-0.2863ln*x* | 0.863 | *y*=0.13-4e-04*x* | 0.999 |
| 115 | *y*=256.33*x*^-0.89^ | 0.999 | *y*=0.39-0.0010*x* | 0.890 | *y*=0.39-0.0010*x* | 0.890 |
| 116 | *y*=165.42*x*^-0.75^ | 0.986 | *y*=20.24*x*^-0.7486^ | 0.994 | *y*=0.19e^0.0077^*^x^* | 1.000 |
| 117 | *y*=9.49-0.05*x* | 0.917 | *y*=3.15-0.587ln*x* | 0.932 | *y*=0.27-0.0349ln*x* | 0.853 |
| 118 | *y*=876.86*x*^-1.24^ | 0.993 | *y*=3.99-0.7600ln*x* | 0.999 | *y*=0.72-0.0995ln*x* | 1.000 |
| 119 | *y*=2670.25*x*^-1.53^ | 0.995 | *y*=1.09e^-0.0081^*^x^* | 0.999 | *y*=0.13e^0.0112^*^x^* | 0.995 |
| 120 | *y*=9.93-0.07*x* | 0.850 | *y*=0.75-0.0045*x* | 0.974 | *y*=0.15e^0.0087^*^x^* | 0.960 |
| 121 | *y*=12.43-0.08*x* | 0.958 | *y*=3.03-0.5786ln*x* | 0.992 | *y*=0.29-7e-04*x* | 0.976 |
| 122 | *y*=21.19e^-0.01^*^x^* | 0.942 | *y*=4.24-0.7957ln*x* | 0.977 | *y*=2.01*x*^-0.3990^ | 1.000 |
| 123 | *y*=1171.05*x*^-1.56^ | 0.933 | *y*=1.54e^-0.0139^*^x^* | 0.996 | *y*=2.20*x*^-0.5899^ | 0.989 |
| 124 | *y*=29.68-5.78ln*x* | 0.983 | *y*=2.73*x*^-0.2800^ | 0.928 | *y*=0.25-0.0011*x* | 0.927 |
| 125 | *y*=28.42-0.13*x* | 0.933 | *y*=10.02*x*^-0.4456^ | 0.994 | *y*=0.58-0.0014*x* | 0.954 |
| 126 | *y*=26.85-0.14*x* | 0.989 | *y*=3.25-0.5115ln*x* | 0.994 | *y*=1.03e^-0.0041^*^x^* | 0.970 |
| 127 | *y*=32.53-0.14*x* | 0.925 | *y*=1.73-0.0016*x* | 0.852 | *y*=1.90-0.2177ln*x* | 0.989 |
| 128 | *y*=85.02*x*^-0.54^ | 0.893 | *y*=1.94-0.0184*x* | 0.970 | *y*=3.46*x*^-0.5447^ | 0.979 |
| 129 | *y*=520.13*x*^-1.07^ | 0.995 | *y*=4.40*x*^-0.3200^ | 0.936 | *y*=1.43-0.2129ln*x* | 0.968 |
| 130 | *y*=3.49-0.02*x* | 0.998 | *y*=5.69-1.0634ln*x* | 1.000 | y=0.90-0.0015*x* | 0.889 |
| 131 | *y*=22.36e^-0.03^*^x^* | 0.990 | *y*=1.08-0.0086*x* | 0.988 | y=0.61-0.1219ln*x* | 0.878 |
| 132 | *y*=114.19*x*^-1.05^ | 0.945 | *y*=14.25*x*^-1.0179^ | 0.900 | *y*=-0.72+0.3141ln*x* | 0.951 |
| 133 | *y*=8.90*x*^-0.25^ | 0.864 | *y*=1.45-0.2461ln*x* | 0.998 | *y*=0.33e^-0.0107^*^x^* | 0.944 |
| 134 | *y*=8.57e^-0.01^*^x^* | 0.984 | *y*=1.36-0.1776ln*x* | 0.863 | *y*=0.28-5e-04*x* | 0.919 |
| 135 | *y*=20.12e^-0.05^*^x^* | 1.000 | *y*=2.44-0.5639ln*x* | 1.000 | *y*=3.22*x*^-0.8138^ | 0.968 |
| 136 | *y*=22.18-4.11ln*x* | 0.870 | *y*=1.79-0.3172ln*x* | 0.876 | *y*=51.44*x*^-1.1319^ | 0.988 |
| 137 | *y*=5.78-1.08ln*x* | 0.996 | *y*=0.52*x*^-0.2149^ | 0.924 | *y*=-0.33+0.1646ln*x* | 0.997 |
| 138 | *y*=44.62*x*^-0.46^ | 0.933 | *y*=4.40*x*^-0.4565^ | 1.000 | *y*=0.41+0.0756ln*x* | 0.957 |
| 139 | *y*=14.18-1.92ln*x* | 0.997 | *y*=0.95-0.0042*x* | 0.999 | *y*=0.78e^0.0031^*^x^* | 0.997 |
| 140 | *y*=19.81*x*^-0.38^ | 0.985 | *y*=1.74-0.3356ln*x* | 0.998 | *y*=0.59e^0.0043^*^x^* | 0.978 |
| 141 | *y*=41.69-0.32*x* | 0.980 | *y*=12.26e^-0.0308^*x* | 1.000 | *y*=1.77-0.0128*x* | 0.993 |
| 142 | *y*=18.61e^-0.01^*^x^* | 1.000 | *y*=6.96*x*^-0.5154^ | 0.934 | *y*=0.75-0.0038*x* | 1.000 |
| 143 | *y*=26.34e^-0.02^*^x^* | 1.000 | *y*=339.91*x*^-1.5606^ | 0.977 | *y*=0.34e^-0.0106^*^x^* | 0.998 |
| 144 | *y*=41.53*x*^-0.44^ | 0.919 | *y*=5.25x^-0.5404^ | 1.000 | *y*=0.94*x*^-0.1344^ | 0.999 |
| 145 | *y*=8.12-0.04*x* | 0.945 | *y*=1.03-0.0048*x* | 0.993 | *y*=0.91e^0.0015^*^x^* | 0.997 |
| 146 | y=3.78-0.01*x* | 0.889 | *y*=0.57-0.0041*x* | 0.942 | *y*=0.62e^0.0047^*^x^* | 0.901 |
| 147 | *y*=15.66*x*^-0.25^ | 1.000 | *y*=1.81*x*^-0.3741^ | 0.982 | *y*=1.17e^0.0036^*^x^* | 0.957 |
| 148 | *y*=11.42-0.10*x* | 0.991 | *y*=1.10-0.0092*x* | 1.000 | *y*=1.86-0.0139*x* | 0.864 |
| 149 | *y*=16.82-3.14ln*x* | 0.999 | *y*=1.22e^-0.0157^*^x^* | 0.999 | *y*=0.98e^0.0035^*^x^* | 0.880 |
| 150 | *y*=14.67-2.62ln*x* | 0.981 | *y*=1.11-0.0072*x* | 0.956 | *y*=1.41-0.0021*x* | 0.893 |
| 151 | *y*=9.23-0.05*x* | 0.980 | *y*=1.58-0.2606ln*x* | 0.950 | *y*=0.66-0.0018*x* | 0.975 |
| 152 | *y*=35.10*x*^-0.47^ | 0.987 | *y*=0.99e^-0.0098^*^x^* | 0.995 | *y*=0.95-0.0059*x* | 0.980 |
| 153 | *y*=4.52e^-0.02^*^x^* | 0.943 | *y*=20.61*x*^-1.1900^ | 0.959 | *y*=0.90-0.1697ln*x* | 0.949 |
| 154 | *y*=19.41-2.93ln*x* | 0.995 | *y*=1.31-0.0061*x* | 0.987 | *y*=0.87-0.0063*x* | 0.922 |
| 155 | *y*=3.45e^-0.005^*^x^* | 0.997 | *y*=0.43e-0.0052*x* | 0.999 | *y*=0.59-0.0021*x* | 0.975 |
| 156 | *y*=14.71*x*^-0.33^ | 0.970 | *y*=1.23-0.1679ln*x* | 0.988 | *y*=-0.22+0.16ln*x* | 0.922 |
| 157 | *y*=11.42-1.84ln*x* | 0.980 | *y*=1.31-0.2071ln*x* | 0.922 | *y*=0.41e^0.0039^*^x^* | 0.899 |
| 158 | *y*=4.14-0.01*x* | 0.873 | *y*=0.75e^-0.0220^*^x^* | 0.999 | *y*=0.49+0.0181ln*x* | 0.979 |
| 159 | *y*=12.25-2.46ln*x* | 0.979 | *y*=0.85-0.0071*x* | 0.994 | *y*=0.52e^0.0064^*^x^* | 0.977 |
| 160 | *y*=10.28-0.04*x* | 0.991 | *y*=0.94-0.0023*x* | 0.858 | *y*=0.96-0.0024*x* | 0.939 |
| 161 | *y*=22.45-3.83ln*x* | 0.954 | *y*=7.62*x*^-0.6392^ | 0.985 | *y*=0.17*x*^0.2854^ | 0.999 |
| 162 | *y*=19.42e^-0.01^*^x^* | 0.873 | *y*=1.86e^-0.0110^*^x^* | 0.909 | *y*=0.41e^0.0062^*^x^* | 0.987 |
| 163 | *y*=22.60-0.10*x* | 0.963 | *y*=1.64-0.0062*x* | 0.948 | *y*=0.45e^0.0024^*^x^* | 0.960 |
| 164 | *y*=364.60*x*^-1.04^ | 0.934 | *y*=2.95-0.5093ln*x* | 0.992 | *y*=0.22e^0.0080^*^x^* | 0.990 |
| 165 | *y*=30.67-5.94ln*x* | 0.988 | *y*=5.12*x*^-0.5124^ | 0.984 | *y*=1.32-0.1905ln*x* | 0.872 |
| 166 | *y*=56.02-10.76ln*x* | 0.996 | *y*=4.25-0.7675ln*x* | 0.959 | *y*=0.17e^0.0110^*^x^* | 0.987 |
| 167 | *y*=19.71e^-0.01^*^x^* | 0.994 | *y*=1.97e^-0.0105^*^x^* | 0.959 | *y*=0.660.0020*x* | 0.944 |
| 168 | *y*=1073.62*x*^-1.34^ | 0.934 | *y*=1.95e^-0.0124^*^x^* | 0.979 | *y*=0.71-0.0039*x* | 0.986 |
| 169 | *y*=19.67-4.14ln*x* | 0.974 | *y*=0.98-0.0072*x* | 0.983 | *y*=0.01*x*^1.1099^ | 0.982 |
| 170 | *y*=29.15-0.21*x* | 0.857 | *y*=2.55-0.0182*x* | 0.976 | *y*=0.97-0.0028*x* | 0.998 |
| 171 | *y*=31.13-5.86ln*x* | 1.000 | *y*=4.15-0.8056ln*x* | 1.000 | *y*=0.53e^0.0089^*^x^* | 0.989 |
| 172 | *y*=15.74-0.08*x* | 0.852 | *y*=1.27e^-0.0154^*^x^* | 0.995 | *y*=1.66e^-0.0052^*^x^* | 0.994 |
| 173 | *y*=9.94-0.04*x* | 0.933 | *y*=1.21-0.1331ln*x* | 0.997 | *y*=1.37-0.0064*x* | 0.963 |
| 174 | *y*=15.19-2.28ln*x* | 1.000 | *y*=1.96*x*^-0.3181^ | 0.996 | *y*=348.68*x*^-1.8792^ | 0.998 |
| 175 | *y*=8.88-0.05*x* | 0.956 | *y*=0.95e^-0.0090^*^x^* | 0.854 | *y*=0.35e^0.0082^*^x^* | 0.979 |
| 176 | *y*=5.17-0.02*x* | 0.925 | *y*=0.64-0.0034*x* | 0.880 | *y*=0.01*x*^0.8395^ | 0.953 |
| 177 | *y*=16.91-2.73ln*x* | 0.865 | *y*=1.86-0.3181ln*x* | 0.851 | *y*=0.87-0.0040*x* | 0.992 |
| 178 | *y*=11.25-1.99ln*x* | 0.871 | *y*=1.47-0.2539ln*x* | 0.867 | *y*=0.68e^-0.0036^*^x^* | 0.988 |
| 179 | *y*=7.81e^-0.01^*^x^* | 0.990 | *y*=0.90-0.0040*x* | 0.973 | *y*=0.44e^0.0036^*^x^* | 0.920 |
| 180 | *y*=13.63-2.17ln*x* | 0.998 | *y*=2.77*x*^-0.4240^ | 0.970 | *y*=1.04-0.0041*x* | 0.983 |
| 181 | *y*=25.44*x*^-0.33^ | 0.999 | *y*=2.87-0.3730ln*x* | 0.994 | *y*=0.82e^0.0044^*^x^* | 1.000 |
| 182 | *y*=10.42-0.08*x* | 0.991 | *y*=2.67-0.4488ln*x* | 0.975 | *y*=2.16-0.3546ln*x* | 0.977 |
| 183 | *y*=9.88-0.05*x* | 0.958 | *y*=0.89-0.0029*x* | 0.932 | *y*=0.64-0.0019*x* | 0.936 |
| 184 | *y*=15.36*x*^-0.11^ | 0.982 | *y*=1.44e-0.0040*x* | 0.988 | *y*=0.83-0.0015*x* | 0.879 |
| 185 | *y*=14.14-2.46ln*x* | 0.999 | *y*=0.88-0.0043*x* | 0.987 | *y*=0.45e0.0070*x* | 0.975 |
| 186 | *y*=37.26-6.33ln*x* | 0.896 | *y*=3.03-0.4557ln*x* | 0.893 | *y*=4.83*x*-0.5041 | 0.972 |
| 187 | *y*=11.66e^-0.01^*^x^* | 0.997 | *y*=1.01-0.0060x | 0.992 | *y*=0.59e0.0108*x* | 0.975 |
| 188 | *y*=29.77e^-0.02^*^x^* | 0.932 | *y*=2.61e^-0.0204^*^x^* | 0.919 | *y*=1.98-0.0186*x* | 0.869 |
| 189 | *y*=44.28e^-0.01^*^x^* | 0.889 | *y*=0.07+0.0124*x* | 0.942 | *y*=2.06-0.3779ln*x* | 0.929 |
| 190 | *y*=9.13-0.07*x* | 0.997 | *y*=0.57e^0.0093^*^x^* | 0.983 | *y*=0.13*x*^0.6433^ | 0.990 |
| 191 | *y*=4569.95*x*^-1.48^ | 0.952 | *y*=592.23*x*^-1.6126^ | 0.991 | *y*=84.64*x*^-1.1227^ | 0.926 |
| 192 | *y*=81.69-1.01*x* | 0.989 | *y*=-1.04+0.0563*x* | 0.974 | *y*=0.41e^0.0216^*^x^* | 0.972 |
| 193 | *y*=109.44-22.31ln*x* | 0.982 | *y*=-0.02+0.0154*x* | 0.965 | *y*=1.69e^0.0023^*^x^* | 0.949 |
| 194 | *y*=148.66*x*^-0.87^ | 0.889 | *y*=18.12*x*^-0.8994^ | 0.951 | *y*=1.12-0.0056*x* | 0.983 |
| 195 | *y*=18.65e^-0.02^*^x^* | 0.987 | *y*=31.79*x*^-1.0933^ | 0.985 | *y*=0.30*x*^0.2332^ | 0.993 |
| 196 | *y*=27.52-5.07ln*x* | 0.919 | *y*=2.56-0.4538ln*x* | 0.902 | *y*=0.89-0.0037*x* | 0.966 |
| 197 | *y*=42.31*x*^-0.50^ | 0.863 | *y*=4.22*x*^-0.4962^ | 0.898 | *y*=0.38+0.0103*x* | 0.982 |
| 198 | *y*=8.30e^-0.01^*^x^* | 0.913 | *y*=0.71-0.0038*x* | 0.931 | *y*=1.60+-0.0078*x* | 0.992 |
| 199 | *y*=13.75*x*^-0.32^ | 0.989 | *y*=1.51*x*-0.3809 | 0.999 | *y*=0.98-0.0656ln*x* | 1.000 |
| 200 | *y*=6.47*x*^-0.13^ | 0.926 | *y*=2.43*x*-0.5970 | 0.863 | *y*=0.54e^0.0097^*^x^* | 1.000 |
| 201 | *y*=23.60-4.30ln*x* | 0.960 | *y*=6.87*x*^-0.6491^ | 0.987 | *y*=0.89-0.0034*x* | 0.988 |
| 202 | *y*=23.04*x*^-0.42^ | 0.968 | *y*=1.75-0.2903ln*x* | 0.896 | *y*=0.20-5e-04*x* | 0.899 |
| 203 | *y*=457.32x^-1.13^ | 0.981 | *y*=34.43*x*^-1.0102^ | 0.987 | *y*=1.13-0.0054*x* | 0.862 |
| 204 | *y*=5.30e^0.01^*^x^* | 1.000 | *y*=0.56-0.0043*x* | 0.985 | *y*=1.20x^-0.1303^ | 0.953 |
| 205 | *y*=64.91*x*^-0.39^ | 0.961 | *y*=2.91*x*-0.0964 | 0.973 | *y*=0.73-0.0061*x* | 0.983 |
| 206 | *y*=89.99*x*^-0.80^ | 0.981 | *y*=2.07-0.3942ln*x* | 0.996 | *y*=0.43-0.0013*x* | 0.898 |
| 207 | *y*=17.01-0.10*x* | 0.999 | *y*=0.23-8e-04*x* | 0.973 | *y*=0.39-0.0364ln*x* | 1.000 |
| 208 | *y*=33.29-0.04*x* | 0.978 | *y*=0.13e^0.0256^*^x^* | 0.997 | *y*=0.49*x*-0.2273 | 0.867 |
| 209 | *y*=41.28-8.27ln*x* | 1.000 | *y*=18.12*x*^-0.8743^ | 0.991 | *y*=0.39-0.0226ln*x* | 0.906 |
| 210 | *y*=17.47-0.08*x* | 0.949 | *y*=3.38*x*^-0.3002^ | 0.941 | *y*=0.49e^0.0054^*^x^* | 0.988 |
| 211 | *y*=159.65*x*^-0.89^ | 1.000 | *y*=4.30*x*^-0.3973^ | 0.976 | *y*=1.31-0.2351ln*x* | 0.984 |
| 212 | *y*=72.01*x*^-0.62^ | 1.000 | *y*=20.82*x*^-0.9576^ | 0.989 | *y*=0.79-0.0017*x* | 0.953 |
| 213 | *y*=131.87*x*^-0.78^ | 0.950 | *y*=27.33*x*^-1.0397^ | 0.985 | *y*=0.49-0.0280ln*x* | 0.859 |
| 214 | *y*=1225.27*x*^-1.51^ | 0.999 | *y*=16.39*x*^-0.8599^ | 0.999 | *y*=1.51*x*^-0.3379^ | 0.931 |
| 215 | *y*=281.83*x*^-0.89^ | 0.976 | *y*=4.06-0.7101ln*x* | 0.945 | *y*=1.06-0.0043*x* | 0.956 |
| 216 | *y*=489.41*x*^-1.03^ | 0.961 | *y*=3.11e^-0.0176^*^x^* | 0.975 | *y*=0.21e^0.0091^*^x^* | 0.996 |
| 217 | *y*=40.48*x*^-0.68^ | 0.998 | *y*=5.80*x*^-0.7698^ | 0.997 | *y*=1.24*x*^-0.3536^ | 0.941 |
| 218 | *y*=66.47*x*^-0.53^ | 0.995 | *y*=-1.62+0.6736ln*x* | 0.991 | *y*=6.60*x*^-0.7731^ | 0.993 |
| 219 | *y*=25.07e^-0.02^*^x^* | 0.975 | *y*=-1.14+0.5448ln*x* | 0.964 | *y*=1.20-0.2281ln*x* | 0.997 |
| 220 | *y*=63.52-12.96ln*x* | 0.996 | *y*=2.06-0.0155*x* | 0.858 | *y*=0.46-0.0025*x* | 0.911 |
| 221 | *y*=92.86*x*^-0.69^ | 0.930 | *y*=2.93*x*^-0.2307^ | 0.868 | *y*=1.10-0.1830ln*x* | 0.986 |
| 222 | *y*=14.13-0.12*x* | 1.000 | *y*=1.35e^-0.0052^*^x^* | 0.995 | *y*=0.50-0.0906ln*x* | 1.000 |
| 223 | *y*=277.28*x*^-0.57^ | 0.940 | *y*=-3.07+1.2219ln*x* | 0.999 | *y*=1.20-0.0031*x* | 0.967 |
| 224 | *y*=122.65*x*^-0.69^ | 0.998 | *y*=3.47-0.5698ln*x* | 0.999 | *y*=1.59-0.2543ln*x* | 1.000 |
| 225 | *y*=27.28e^-0.01^*^x^* | 0.969 | *y*=2.31-0.0162*x* | 0.982 | *y*=8.86*x*^-0.9959^ | 0.957 |
| 226 | *y*=60.34*x*^-0.45^ | 0.898 | *y*=4.69*x*^-0.3719^ | 0.894 | *y*=0.70-0.0035*x* | 0.937 |
| 227 | *y*=24.64*x*^-0.34^ | 0.972 | *y*=6.36*x*^-0.7556^ | 0.983 | *y*=0.18-2e-04*x* | 0.996 |
| 228 | *y*=122.52*x*^-0.91^ | 0.981 | *y*=5.97*x*^-0.5143^ | 0.895 | *y*=0.54e^-0.0021^*^x^* | 0.989 |
| 229 | *y*=254.36*x*^-1.19^ | 0.997 | *y*=20.65*x*^-1.0530^ | 0.984 | *y*=0.03+0.0470ln*x* | 0.999 |
| 230 | *y*=40.41*x*^-0.69^ | 0.985 | *y*=4.23*x*^-0.7327^ | 0.999 | *y*=0.21*x*^-0.3007^ | 0.915 |
| 231 | *y*=10.09-0.08*x* | 1.000 | *y*=1.47-0.0059*x* | 0.949 | *y*=0.94+0.0599ln*x* | 0.957 |
| 232 | *y*=30.54*x*^-0.65^ | 1.000 | *y*=0.89*x*^-0.2033^ | 1.000 | *y*=0.79-0.1525ln*x* | 1.000 |
| 233 | *y*=47.44*x*^-0.50^ | 0.891 | *y*=7.08*x*^-0.5907^ | 0.921 | *y*=1.16*x*^-0.3220^ | 0.867 |
| 234 | *y*=31.54e^-0.07^*^x^* | 0.996 | *y*= 12.61*x*^-0.9176^ | 0.946 | *y*=0.35-0.0059*x* | 1.000 |
| 235 | *y*=14.21e^-0.03^*^x^* | 0.999 | *y*=0.85+-0.0089*x* | 0.999 | *y*=-0.28+0.20ln*x* | 0.989 |
| 236 | *y*=16.64-2.93ln*x* | 1.000 | *y*=1.56*x*^-0.2385^ | 0.908 | *y*=3.09*x*^-0.3870^ | 1.000 |
| 237 | *y*=97.27*x*^-0.75^ | 0.938 | *y*=8.42*x*^-0.7646^ | 0.940 | *y*=0.57*x*^-0.3581^ | 0.997 |
| 238 | *y*=34.54*x*^-0.23^ | 0.925 | *y*=3.08*x*^-0.1970^ | 0.879 | *y*=1.39-0.2682ln*x* | 0.999 |
| 239 | *y*=27.56-4.73ln*x* | 1.000 | *y*=1.26-0.0084*x* | 0.998 | *y*=4.08*x*^-0.7808^ | 0.995 |
| 240 | *y*=91.85*x*^-0.91^ | 0.946 | *y*=7.80*x*^-0.9554^ | 0.964 | *y*=3.46*x*^-0.5203^ | 0.909 |
| 241 | *y*=257.17*x*^-0.98^ | 0.959 | *y*=9.45*x*^-0.6024^ | 0.996 | *y*=0.49-0.0849ln*x* | 0.960 |
| 242 | *y*=204.59*x*^-0.87^ | 0.999 | *y*=10.72*x*^-0.7246^ | 1.000 | *y*=0.78*x*^-0.2505^ | 0.917 |
| 243 | *y*=95.45*x*^-0.76^ | 0.980 | *y*=3.29*x*^-0.2737^ | 0.905 | *y*=0.41-0.0017*x* | 0.933 |
| 244 | *y*=20.63e^-0.02^*^x^* | 0.997 | *y*=-0.82+0.4798ln*x* | 0.979 | *y*=0.82-0.1504ln*x* | 0.970 |
| 245 | *y*=27.53e^-0.01^*^x^* | 0.994 | *y*=0.04*x*^0.7683^ | 0.895 | *y*=0.13e^0.0186^*^x^* | 0.980 |
| 246 | *y*=175.86*x*^-0.95^ | 0.935 | *y*=10.48*x*^-0.7536^ | 0.959 | *y*=0.47e^-0.0278^*^x^* | 0.992 |
| 247 | *y*=26.89*x*^-0.55^ | 0.989 | *y*=1.64*x*^-0.4031^ | 0.861 | *y*=0.61*x*^-0.2570^ | 0.999 |
| 248 | *y*=89.53*x*^-0.62^ | 0.963 | *y*=5.97*x*^-0.5214^ | 0.939 | *y*=1.54e^-0.0032^*^x^* | 0.993 |
| 249 | *y*=41.28-7.50ln*x* | 1.000 | *y*=0.37*x*^0.3144^ | 0.850 | *y*=0.61-0.0017*x* | 0.869 |
| 250 | *y*=34.74*x*^-0.30^ | 0.952 | *y*=10.28*x*-0.7619 | 0.997 | *y*=0.47*x*-0.0909 | 0.982 |
| 251 | *y*=102.03*x*^-0.70^ | 0.997 | *y*=7.84*x*-0.6321 | 0.963 | *y*=0.29+0.0182lnx | 0.992 |
| 252 | *y*=204.07*x*^-0.93^ | 0.937 | *y*=20.58*x*-0.9434 | 0.940 | *y*=0.71-0.0030*x* | 0.906 |
| 253 | *y*=23.97*x*^-0.53^ | 0.936 | *y*=1.38-0.2143ln*x* | 1.000 | *y*=0.56*x*-0.3185 | 1.000 |
| 254 | *y*=130.44*x*^-0.88^ | 0.857 | *y*=7.74*x*-0.6590 | 0.884 | *y*=0.44-0.0041*x* | 0.996 |
| 255 | *y*=434.55*x*^-1.14^ | 1.000 | *y*=14.97*x*^-0.8139^ | 0.957 | *y*=0.65*x*^-0.1457^ | 0.932 |
| 256 | *y*=505.24*x*^-1.19^ | 0.956 | *y*=128.57*x*^-1.4833^ | 0.968 | *y*=0.33+0.0011*x* | 0.999 |
| 257 | *y*=1152.55e^-0.08^*^x^* | 1.000 | *y*=15.18-3.49ln*x* | 0.958 | *y*=0.66-0.0065*x* | 1.000 |
| 258 | *y*=31.31-6.10ln*x* | 0.915 | *y*=3.24-0.5998ln*x* | 0.919 | *y*=0.95-0.1262ln*x* | 0.889 |
| 259 | *y*=145.78*x*^-0.52^ | 0.988 | *y*=-3.76+1.26ln*x* | 0.887 | *y*=2.71*x*^-0.3817^ | 0.996 |
| 260 | *y*=164.05*x*^-0.85^ | 0.995 | *y*=8.34*x*^-0.6243^ | 0.960 | *y*=1.07*x*^-0.3230^ | 0.982 |
| 261 | *y*=22.33e^-0.01^*^x^* | 0.981 | *y*=3.72*x*^-0.1791^ | 0.975 | *y*=0.73-0.0061*x* | 0.983 |
| 262 | *y* =52.45*x*^-0.61^ | 0.869 | *y*=7.06*x*^-0.6541^ | 0.943 | *y*=1.69*x*^-0.4456^ | 0.872 |
| 263 | *y*=21.59e^-0.005^*^x^* | 0.999 | *y*=2.69-0.4012ln*x* | 0.997 | *y*=0.41+0.0502ln*x* | 0.972 |
| 264 | *y*=35.88-6.44ln*x* | 0.999 | *y*=1.81e^-0.0119^*^x^* | 1.000 | *y*=0.36-8e-04*x* | 0.983 |
| 265 | *y*=67.59*x*^-0.47^ | 0.986 | *y*=4.52*x*^-0.3252^ | 0.884 | *y*=2.12*x*^-0.0433^ | 0.891 |
| 266 | *y*=120.18*x*^-0.72^ | 0.957 | *y*=0.07+0.0151*x* | 0.994 | *y*=1.29*x*^-0.2434^ | 0.999 |
| 267 | *y*=191.49*x*^-0.92^ | 0.908 | *y*=3.05*x*^-0.3537^ | 0.975 | *y*=1.53-0.2218ln*x* | 0.999 |
| 268 | *y*=94.94*x*^-0.97^ | 0.946 | *y*=5.82*x*^-0.8811^ | 0.919 | *y*=1.83*x*^-0.9661^ | 0.991 |
| 269 | *y*=36.51-7.29ln*x* | 0.999 | *y*=11.89*x*^-0.7620^ | 0.982 | *y*=0.26*x*^-0.0423^ | 0.887 |
| 270 | *y*=46.48-0.85*x* | 0.912 | *y*=0.08e^0.0627^*^x^* | 0.932 | *y*=2.51*x*^-0.1383^ | 1.000 |
| 271 | *y*=1279.72*x*^-1.39^ | 0.864 | *y*=297.71*x*^-1.6669^ | 0.853 | *y*=1.05-0.2003ln*x* | 0.923 |
| 272 | *y*=57.82-0.74*x* | 0.997 | *y*=0.40-0.0017*x* | 0.951 | *y*=0.44+0.0499ln*x* | 0.950 |
| 273 | *y*=16.11-0.13*x* | 0.872 | *y*=2.26-0.0207*x* | 0.989 | *y*=0.16*x*^0.1833^ | 0.944 |
| 274 | *y*=189.87*x*^-0.78^ | 0.986 | *y*=28.29*x*^-0.8972^ | 0.912 | *y*=0.35e^0.0077^*^x^* | 0.990 |
| 275 | *y*=59.49*x*^-0.49^ | 1.000 | *y*=1.30-0.0098*x* | 0.999 | *y*=0.59*x*-0.4358 | 0.982 |
| 276 | *y*=31.11e^-0.03^*^x^* | 0.996 | *y*=3.29-0.6458ln*x* | 0.998 | *y*=0.63-0.0047*x* | 0.997 |
| 277 | *y*=19.45*x*^-0.16^ | 0.943 | *y*=2.43*x*^-0.2055^ | 0.999 | *y*=0.80*x*-^0.0921^ | 0.929 |
| 278 | *y*=124.07*x*^-0.96^ | 0.964 | *y*=17.60*x*^-1.0239^ | 1.000 | *y*=0.96*x*^-0.3754^ | 0.890 |
| 279 | *y*=8.61-0.07*x* | 1.000 | *y*=0.82-0.0040*x* | 0.997 | *y*=0.38e^-0.0124^*^x^* | 1.000 |
| 280 | *y*=74.81*x*^-0.85^ | 0.997 | *y*=1.12e^-0.0219^*^x^* | 0.999 | *y*=1.48*x*^-0.6352^ | 0.966 |
| 281 | *y*=48.36-10.22ln*x* | 0.993 | *y*=2.01-0.0213*x* | 0.996 | *y*=0.40-9e-04*x* | 0.978 |
| 282 | *y*=17.90+-0.12*x* | 1.000 | *y*=1.78-0.0111*x* | 1.000 | *y*=-0.13+0.24ln*x* | 0.996 |
| 283 | *y*=11.41e^-0.01^*^x^* | 1.000 | *y*=4.07*x*^-0.4330^ | 1.000 | *y*=0.43-9e-04*x* | 1.000 |
| 284 | *y*=39.43-7.37ln*x* | 0.853 | *y*=3.69-0.6207lnx | 0.860 | *y*=0.56-0.0014*x* | 0.897 |
| 285 | *y* =24.18-4.56ln*x* | 0.864 | *y*=2.31-0.4397ln*x* | 0.990 | *y*=0.27e^0.0166^*^x^* | 0.993 |
| 286 | *y* = 16.77e^-0.03^*^x^* | 0.996 | *y*=2.33-0.4479ln*x* | 1.000 | *y*=0.23e^0.0097^*^x^* | 0.981 |
| 287 | *y*=580.30*x*^-1.28^ | 0.976 | *y*=17.17*x*^-0.8796^ | 0.970 | *y*=0.37e^0.0076^*^x^* | 0.983 |
| 288 | *y*=4.40-0.03*x* | 0.897 | *y*=0.70*x*^-0.2081^ | 0.935 | *y*=0.62*x*^-0.1883^ | 0.913 |
| 289 | *y*=16.37-0.08*x* | 0.947 | *y*=1.86-0.0163*x* | 0.849 | *y*=0.53*x*^-0.0696^ | 0.876 |
| 290 | *y*=23.70e^-0.01^*^x^* | 0.999 | *y*=3.16-0.4619ln*x* | 0.995 | *y*=0.53-0.0028*x* | 0.989 |
| 291 | *y* =82.99-18.66ln*x* | 0.852 | *y*=-0.004+0.0191*x* | 0.881 | *y*=0.48-0.0016*x* | 0.912 |
| 292 | *y*=16.08e^-0.02^*^x^* | 0.960 | *y*=1.18-0.0030*x* | 0.917 | *y*=0.24e^0.0063^*^x^* | 0.929 |
| 293 | *y*=26.91e^-0.02^*^x^* | 0.993 | *y*=2.27-0.0201*x* | 0.977 | *y*=0.75-0.0031*x* | 0.991 |
| 294 | *y*=36.28-7.22ln*x* | 0.993 | *y*=3.67-0.7020ln*x* | 0.988 | *y*=0.58-0.0028*x* | 0.900 |
| 295 | *y*=236.98*x*^-1.01^ | 0.996 | *y*=9.65*x*^-0.7432^ | 0.999 | *y*=0.49-0.0666ln*x* | 0.902 |
| 296 | *y*=102.36*x*^-0.67^ | 0.991 | *y*=8.46*x*^-0.5941^ | 0.979 | *y*=0.82-0.0739ln*x* | 0.998 |
| 297 | *y*=156.92*x*^-0.72^ | 0.983 | *y*=13.39*x*^-0.6672^ | 0.977 | *y*=0.68-0.0022*x* | 0.879 |
| 298 | *y*=13.23e^-0.01^*^x^* | 0.929 | *y*=1.13e^-0.0111^*^x^* | 0.960 | *y*=0.70-0.0034*x* | 0.914 |
| 299 | *y*=98.09*x*^-0.83^ | 0.952 | *y*=8.11*x*^-0.7234^ | 0.958 | *y*=0.50e^-0.0054^*^x^* | 0.997 |
| 300 | *y*=17.29-3.46ln*x* | 0.921 | *y*=1.90-0.3737ln*x* | 0.911 | *y*=0.73-0.0044*x* | 0.992 |
| 301 | *y*=15.08-0.14*x* | 0.983 | *y*=1.45-0.0125*x* | 0.980 | *y*=0.56-0.0016*x* | 0.950 |
| 302 | *y*=173.37*x*^-0.62^ | 0.925 | *y*=3.33e^-0.0158^*^x^* | 0.940 | *y*=1.92e^-0.0023^*^x^* | 0.992 |
| 303 | *y*=50.59*x*^-0.36^ | 0.956 | *y*=22.84*x*^-0.8958^ | 1.000 | *y*=17.72*x*^-1.2907^ | 0.871 |
| 304 | *y*=35.39*x*^-0.11^ | 0.890 | *y*=1.84e^0.0036^*^x^* | 0.855 | *y*=0.03*x*^0.8207^ | 0.858 |
| 305 | *y*=20.19-3.81ln*x* | 0.993 | *y*=1.92-0.3451ln*x* | 0.992 | *y*=0.43e^0.0016^*^x^* | 0.981 |

SOC, soil organic carbon; TN, total nitrogen; TP, total phosphorus. *R*^2^: coefficient of determination of the fitted equation.

**Table S4. Variance inflation factor (VIF) analysis between changes in soil C:N and environmental variables.**

| Variable | 0-20 cm | 20-40 cm | 40-60 cm | 60-100 cm |
| --- | --- | --- | --- | --- |
|  | VIF values | VIF values | VIF values | VIF values |
| C input | 1.482 | 1.497 | 1.495 | 1.495 |
| N input | 1.550 | 1.579 | 1.568 | 1.578 |
| P input | 1.536 | 1.528 | 1.530 | 1.528 |
| ΔAET | 1.359 | 1.358 | 1.358 | 1.359 |
| AT | 4.346 | 4.392 | 4.335 | 4.317 |
| AP | 4.964 | 4.096 | 4.105 | 4.053 |
| T_season_ | 4.274 | 4.260 | 4.260 | 4.260 |
| P_season_ | 2.006 | 2.014 | 2.009 | 2.012 |
| pH | 1.425 | 1.815 | 1.812 | 1.812 |
| Sand | 1.891 | 1.900 | 1.901 | 1.902 |
| Clay | 2.003 | 2.041 | 2.040 | 2.042 |
| **ΔBD** | **10.331** | **10.095** | **10.158** | **10.676** |
| **ΔCEC** | **7.566** | **7.532** | **7.913** | **7.025** |
| C:N_1980s_ | 1.179 | 1.184 | 1.133 | 1.135 |

Variables with a variance inflation factor (VIF) greater than 5 were excluded from subsequent analyses to avoid multicollinearity. C input, N input, and P input, the cumulative C, N, and P inputs over the last 40 years, respectively; ΔAET, changes in actual evapotranspiration from 1981–1985 (average) to 2019–2023 (average); AT, accumulated temperature; AP, accumulated precipitation; T_season_, temperature seasonality; P_season_, precipitation seasonality; ΔBD, relative changes in bulk density from the 1980s to 2023; ΔCEC, relative changes in cation exchange capacity from the 1980s to 2023; C:N_1980s_, background (1980s) soil C:N stoichiometry.

**Table S5. Variance inflation factor (VIF) analysis between changes in soil C:P and environmental variables.**

| Variable | 0-20 cm | 20-40 cm | 40-60 cm | 60-100 cm |
| --- | --- | --- | --- | --- |
|  | VIF values | VIF values | VIF values | VIF values |
| C input | 1.483 | 1.501 | 1.497 | 1.496 |
| N input | 1.535 | 1.541 | 1.549 | 1.554 |
| P input | 1.552 | 1.552 | 1.574 | 1.575 |
| ΔAET | 1.359 | 1.360 | 1.359 | 1.363 |
| AT | 4.323 | 4.305 | 4.304 | 4.301 |
| AP | 4.182 | 4.184 | 4.336 | 4.052 |
| T_season_ | 4.329 | 4.394 | 4.355 | 4.308 |
| P_season_ | 2.003 | 2.009 | 2.021 | 2.010 |
| pH | 1.461 | 1.885 | 1.873 | 1.910 |
| Sand | 1.891 | 1.907 | 1.914 | 1.914 |
| Clay | 2.005 | 2.060 | 2.061 | 2.045 |
| **ΔBD** | **10.315** | **10.006** | **10.147** | **10.549** |
| **ΔCEC** | **7.614** | **7.530** | **7.875** | **7.003** |
| C:P_1980s_ | 1.294 | 1.296 | 1.233 | 1.252 |

Variables with a variance inflation factor (VIF) greater than 5 were excluded from subsequent analyses to avoid multicollinearity. C input, N input, and P input, the cumulative C, N, and P inputs over the last 40 years, respectively; ΔAET, changes in actual evapotranspiration from 1981–1985 (average) to 2019–2023 (average); AT, accumulated temperature; AP, accumulated precipitation; T_season_, temperature seasonality; P_season_, precipitation seasonality; ΔBD, relative changes in bulk density from the 1980s to 2023; ΔCEC, relative changes in cation exchange capacity from the 1980s to 2023; C:P_1980s_, background (1980s) soil C:P stoichiometry.

**Table S6. Variance inflation factor (VIF) analysis between changes in soil N:P and environmental variables.**

| Variable | 0-20 cm | 20-40 cm | 40-60 cm | 60-100 cm |
| --- | --- | --- | --- | --- |
|  | VIF values | VIF values | VIF values | VIF values |
| C input | 1.484 | 1.502 | 1.497 | 1.497 |
| N input | 1.543 | 1.565 | 1.568 | 1.586 |
| P input | 1.543 | 1.560 | 1.577 | 1.598 |
| ΔAET | 1.359 | 1.359 | 1.358 | 1.360 |
| AT | 4.307 | 4.299 | 4.304 | 4.301 |
| AP | 4.967 | 4.096 | 4.067 | 4.534 |
| T_season_ | 4.327 | 4.436 | 4.375 | 4.327 |
| P_season_ | 2.003 | 2.014 | 2.025 | 2.020 |
| pH | 1.461 | 1.879 | 1.872 | 1.949 |
| Sand | 1.891 | 1.900 | 1.903 | 1.904 |
| Clay | 2.006 | 2.049 | 2.049 | 2.041 |
| **ΔBD** | **10.301** | **10.515** | **10.603** | **10.671** |
| **ΔCEC** | **7.619** | **7.556** | **7.912** | **7.724** |
| N:P_1980s_ | 1.173 | 1.220 | 1.185 | 1.255 |

Variables with a variance inflation factor (VIF) greater than 5 were excluded from subsequent analyses to avoid multicollinearity. C input, N input, and P input, the cumulative C, N, and P inputs over the last 40 years, respectively; ΔAET, changes in actual evapotranspiration from 1981–1985 (average) to 2019–2023 (average); AT, accumulated temperature; AP, accumulated precipitation; T_season_, temperature seasonality; P_season_, precipitation seasonality; ΔBD, relative changes in bulk density from the 1980s to 2023; ΔCEC, relative changes in cation exchange capacity from the 1980s to 2023; N:P_1980s_, background (1980s) soil N:P stoichiometry.
